# Systematically understanding the immunity leading to CRPC progression

**DOI:** 10.1371/journal.pcbi.1007344

**Published:** 2019-09-10

**Authors:** Zhiwei Ji, Weiling Zhao, Hui-Kuan Lin, Xiaobo Zhou

**Affiliations:** 1 School of Biomedical Informatics, The University of Texas Health science center at Houston, Houston, Texas, United States of America; 2 Department of Cancer Biology, Wake Forest Baptist Medical Center, Wake Forest University, Winston Salem, North Carolina, United States of America; University of Southern California, UNITED STATES

## Abstract

Prostate cancer (PCa) is the most commonly diagnosed malignancy and the second leading cause of cancer-related death in American men. Androgen deprivation therapy (ADT) has become a standard treatment strategy for advanced PCa. Although a majority of patients initially respond to ADT well, most of them will eventually develop castration-resistant PCa (CRPC). Previous studies suggest that ADT-induced changes in the immune microenvironment (mE) in PCa might be responsible for the failures of various therapies. However, the role of the immune system in CRPC development remains unclear. To systematically understand the immunity leading to CRPC progression and predict the optimal treatment strategy *in silico*, we developed a 3D **H**ybrid **M**ulti-**s**cale **M**odel (**HMSM**), consisting of an ODE system and an agent-based model (ABM), to manipulate the tumor growth in a defined immune system. Based on our analysis, we revealed that the key factors (e.g. WNT5A, TRAIL, CSF1, etc.) mediated the activation of PC-Treg and PC-TAM interaction pathways, which induced the immunosuppression during CRPC progression. Our HMSM model also provided an optimal therapeutic strategy for improving the outcomes of PCa treatment.

## Author summary

Prostate cancer (PCa) is the second leading cause of cancer-related deaths in American men. Androgen deprivation therapy (ADT) is the first-line therapy for advanced PCa, yet a significant number of primary PCa patients treated with ADT eventually develop incurable castration-resistant prostate cancer (CRPC). Recent observations suggest that the immunosuppressive microenvironment of PCa might be responsible for the failures of various therapies. However, the role of immune system in CRPC progression is still unclear. To deeply understand the immunity leading to CRPC progression, we developed a unique systems biology approach (HMSM). Based on our analysis, we identified the key molecules (e.g. WNT5A, TRAIL, CSF1, etc.) mediating the communication of PCa and immune cells. Our HMSM system also revealed the optimal therapeutic strategy for PCa treatment. Collectively, our study provides a new insight to study tumor-related immune mechanisms and pave the way for the development of more effective treatments.

## Introduction

Prostate cancer (PCa) is the second leading cause of cancer-related death in American men [1, 2]. Androgen deprivation therapy (ADT) has become standard therapy for the treatment of PCa. Although the majority of patients initially respond well to ADT, most patients will eventually become unresponsive, and the PCas recur within 1–3 years after ADT as castration-resistant prostate cancers (CRPC) [3].

Previous studies have demonstrated that androgen receptor (AR)-mediated signaling pathway plays a central role in CRPC cell survival and growth, constituting an attractive target for therapy [4, 5]. MDV3100 (enzalutamide), an FDA-approved drug, is a well-known AR antagonist that can effectively block androgen binding to AR, thereby preventing AR nuclear translocation and coactivator recruitment [6, 7]. However, prostate cancer treatment with AR antagonists can also acquire resistance through AR mutations [8–10], such as AR splice variants [11] and gene amplification. Therefore, there is an urgent need for the development of new therapeutic strategies. To date, immunotherapy represents an appealing option in prostate cancer treatment [12]. The FDA-approved vaccine (sipeucel-T [13]) and PD-1 inhibitor (e.g., pembrolizumab [14]) have been used to treat advanced PCa in clinical trials. However, recent phase III trials showed multiple failures of immunotherapy in PCa [15–18].

Recent observations suggest that the microenvironment (mE) of PCa is *immunosuppressive*, which appears to be responsible for the failures of various agents targeting the immune system in PCa [15, 19]. Escamila *et al*. found that tumor-associated macrophages (TAM) exert a negative impact on the treatment response of PCa after ADT [20]. ADT induced an increased expression of colony-stimulating factor 1 (**CSF1**) in prostate cancer cells (PCs), leading to a significant enhancement of TAM infiltration. The increased levels of IL10, VEGF, and EGF in TAMs, in turn, promote treatment resistance by enhancing immune suppression and tumor proliferation. Moreover, in the prostate-specific Pten^-/-^ mouse model, Akins and colleagues found that Treg (Regulatory T cell) expansion occurred following ADT and the frequency and function of CD8^+^ T cells (CTLs) increased at the early stage but reduced after a late time point [21, 22]. In this study, we found that WNT5A activated AKT/AR signaling pathway and stimulated the PC proliferation, which might be a new mechanism of PC resistance to ADT. However, the precise cellular targets and the exact molecular mechanism of the immunity leading to CRPC remain unclear. Therefore, systematic understanding of the impact of androgen deprivation on the tumor-associated immune system will help to characterize novel cytokine networks and signal transduction pathways and develop more effective combined therapies for patients with advanced PCa. Taking above studies together, we **hypothesize** that **1)** following ADT, the reactivation of AR signaling in PC cells and the altered immune mE contribute to the development of CRPC; **2)** the communication between immune cells and PCs results in immune suppression and PCa progression; **3)** targeting the immune-PC pathways mediated by cytokines after ADT may prevent CRPC development.

In recent years, some mathematical approaches have been developed to model the tumor growth, angiogenesis, and drug resistance, providing a new perspective way in exploring the molecular mechanisms of cancer treatment resistance [23–26]. Peng *et al*. developed an ODE-based model to characterize the effect of castration on the immune system and to predict the efficacy of combined therapy with ADT and vaccines on PCs [27]. However, the role of the immune system in CRPC progression was rarely studied. Based on the hypothesis described above, we developed a predictive 3D **H**ybrid **M**ulti-**s**cale **M**odel (HMSM) with various types of data for systematically understanding the immunity leading to CRPC progression.

The HMSM model consists of a 3D agent-based model (ABM) and an ordinary differential equations (ODEs) model. The ABM is used for modeling tumor growth, angiogenesis, immune response in the prostate and lymph node compartments, and the ODEs model for dynamics of intracellular signal transduction. The HMSM model integrates key biological events spatially and temporally. Spatially, the simulated mE contains two components: prostate tumor space and lymph node. PCs and TAMs reside in the tumor space for tumor growth and angiogenesis, and CTLs and Tregs home in the lymph node and infiltrate to tumor bed once the initial immune response is activated. Temporally, we modeled the intracellular signaling dynamics (minutes to hours); cell division, apoptosis, migration, and immune infiltration (hours to days); drug response (days to weeks), and CRPC progression and tumor growth (weeks to months). After parameter tuning, the outcomes of our HMSM model in different conditions are fit with the experimental observations. Finally, we use this model to predict the effect of individual and combined treatments with WNT5A neutralization, CSF1R inhibition [20], IL-2 neutralization [22], and EGFR inhibition [28] on the development of CRPC. Our simulation indicates that suppression of Treg expansion with IL-2 antibody and blockade of PC-Treg and PC-TAM interactions appear to re-activate anti-tumor immune responses and to prevent CRPC occurrence. In summary, this study revealed the key cytokines/pathways-induced immunosuppression during CRPC progression and also provided an optimal therapeutic strategy for improving the outcomes of CRPC treatments.

## Results

### Inference of PC-Treg interactions

To model CRPC progression, we first identified the cell-cell interactions between PC and Treg based on the transcriptomic data. We calculated 1) the significantly overexpressed ligands- and receptors-encoded genes from the GEO (Gene Expression Omnibus) dataset GSE38043 [29] and GSE46218 [30]; 2) the directionality of cell-cell communication of ligand-receptor pairs based on the prior information in public databases, such as iRefWeb [31]. The interactions between PC and Treg were mainly inferred from above two GEO datasets using the approach reported previously [31]. The dataset GSE38043 was generated from isolated Treg cells of CRPC patients (3 patients VS. 3 control). Student T-test was used to filter the significantly overexpressed genes with a p-value < 0.05. In total, we filtered 18 ligand genes (e.g., WNT5A) and 26 receptor genes (e.g., DCR2, EGFR, etc.). The dataset GSE46218 was generated from prostate orthotopic xenograft models. We compared the gene expression profiles of castration-resistant prostate cancer and androgen-dependent prostate cancer, and obtained 23 overexpressed ligand-encoded genes and 39 overexpressed receptor-encoded genes from the castration-resistant PCa, such as FZD5, BMP6, TNFSF10 (TRAIL), etc. The calculation procedure was shown in **S1 Fig**. We did further filtration analysis for the identified ligand- and receptor-encoded gene pairs and found potential pairwise interactions between PC and Treg: Treg→WNT5A→PC, and PC→TRAIL→Treg (**S2 Fig**). All of the significantly overexpressed ligand- and receptor-encoding genes were listed in **S1 and S2 Tables**.

**Fig 1:**
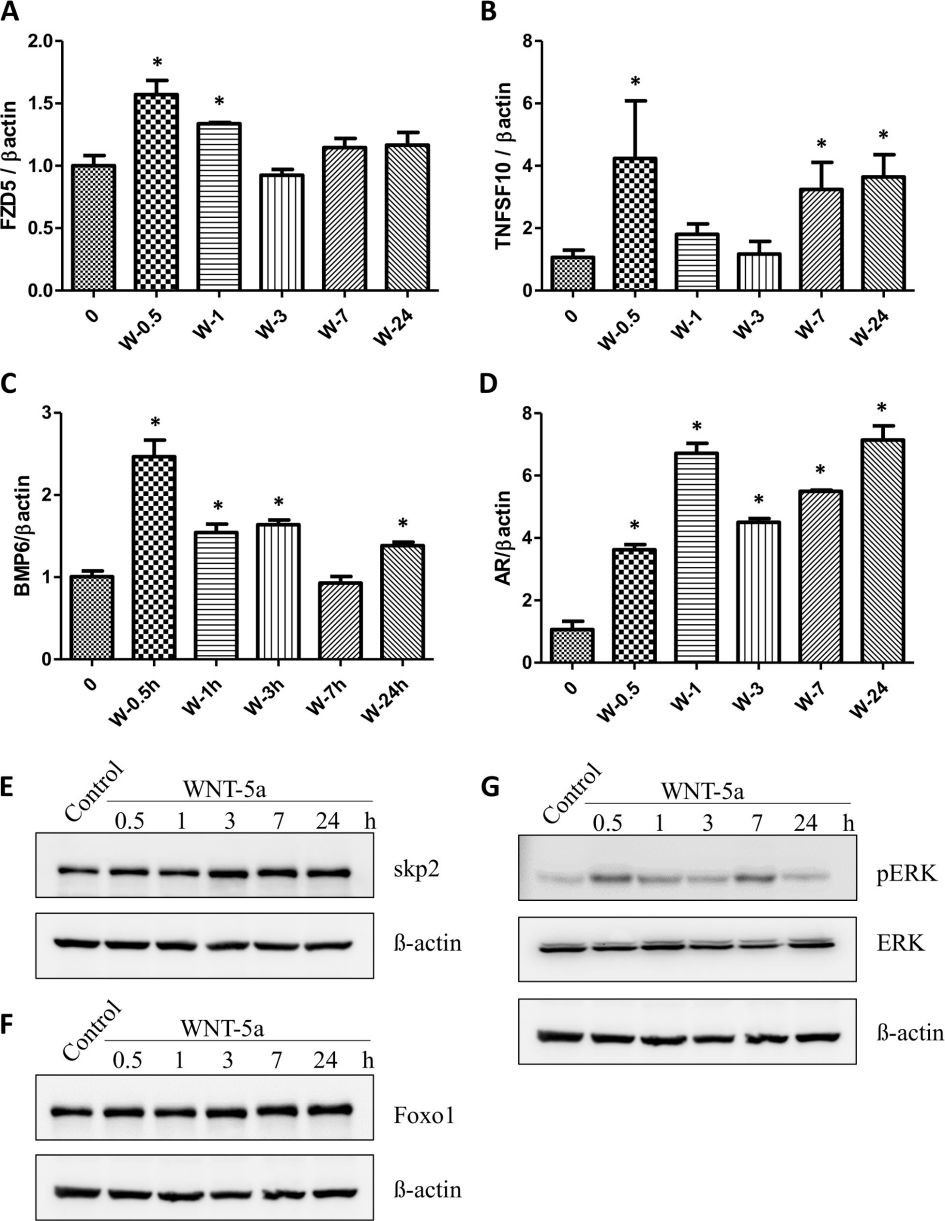
qRT-PCR and Western blot analysis to determine the effect of WNT5A on AR-signaling associated genes and proteins in androgen-resistant 22RV1 prostate cancer cells. (**A-D)** The gene expression levels of FZD5, TNFSF10, BMP6 and AR at 0.5, 1, 3, 7 and 24h after treatment with WNT5A, respectively. (**E-G)** The effect of WNT5A on protein levels of skp2, Foxo1 and pERK, respectively.

To determine the cell-cell interaction inferred above, we treated castration-resistant prostate cancer cells 22RV1 with WNT5A and generated RNA-seq data. Our analysis shows that WNT5A treatment up-regulates a group of genes in 22RV1 cells, e.g., AR, FZD5, SKP2, PKC, ERK, STAT3, and TNFSF10 (TRAIL), etc. (**S3 Fig**). Further functional analysis of the significantly expressed genes shows that some important pathways are enriched, including PI3K/AKT/AR pathway, Ras pathway, MAPK pathway, JAK/STAT pathway, prostate cancer pathway, and WNT pathway, etc (see the details in **S3 Table**). Thus, WNT5A appears to be a key factor in the activation of the survival and proliferation pathways in the castration-resistant PC cells. To further validate the results obtained from RNA-seq analysis and inferred WNT5A/TRAIL pathway loop, we treated 22RV1 cells with WNT5A and the gene expression and protein levels were measured using qRT-PCR and/or Western blot, including FZD5, TNFSF10 (TRAIL), BMP6, AR, BMP6, Skp2, Foxo1, and ERK. WNT5A receptor, FZD5 was significantly up-regulated at 1 hour after treatment (**Fig 1A**). In addition, WNT5A stimulation induced a sharp increase of TNFSF10 (TRAIL) (**Fig 1B**), which may further promote Treg expansion [32]. In addition, WNT5A led to a significant increase in the BMP6 level at 0.5, 1 and 3 hours following treatment (**Fig 1C**). This finding is consistent with the previous studies showing that WNT5A stimulates BMP-6 expression in metastatic prostate cancer (CaP) in the context of bone niche; and BMP-6 in turn stimulated the proliferation of CaP cells [33]. Most important, treating cells with WNT5A resulted in a dramatical and persistent increase in the transcript level of AR (**Fig 1D**). The protein levels of Skp2 and FOXO1 were increased at 3, 7 and 24 hours post-treatment (**Fig 1E and 1F**). These findings are consistent with reports that Skp2 and FOXO1 activation are associated with AR transactivation and tumorigenesis [34, 35]. Finally, increased ERK phosphorylation was observed at 0.5 and 7 hours (**Fig 1G**), also consistent with the early studies that MEK/ERK axis may promote CRPC development, leading to early relapse [36, 37]. Taken together, our experimental results demonstrated that WNT5A induced AR signaling activation and secretion of TRAIL, which potentially promotes CRPC development.

### Inference of PC-TAM interaction

We also identified the PC-TAM interactions based on the previous findings. Escamilla and coworkers found that CSF1 was significantly induced in the prostate cancer cells by ADT, leading to a significant increase in TAM [20]. TAM expresses elevated levels of VEGF, MMP-9, IL10, and EGF, thereby to promote the protumorigenic phenotype (such as angiogenesis and immune suppression) of macrophages [20, 38]. Tang *et al*. reported that Treg expansion in Pten^-/-^ mice after castration was mediated by IL-2 [22].

**Fig 2:**
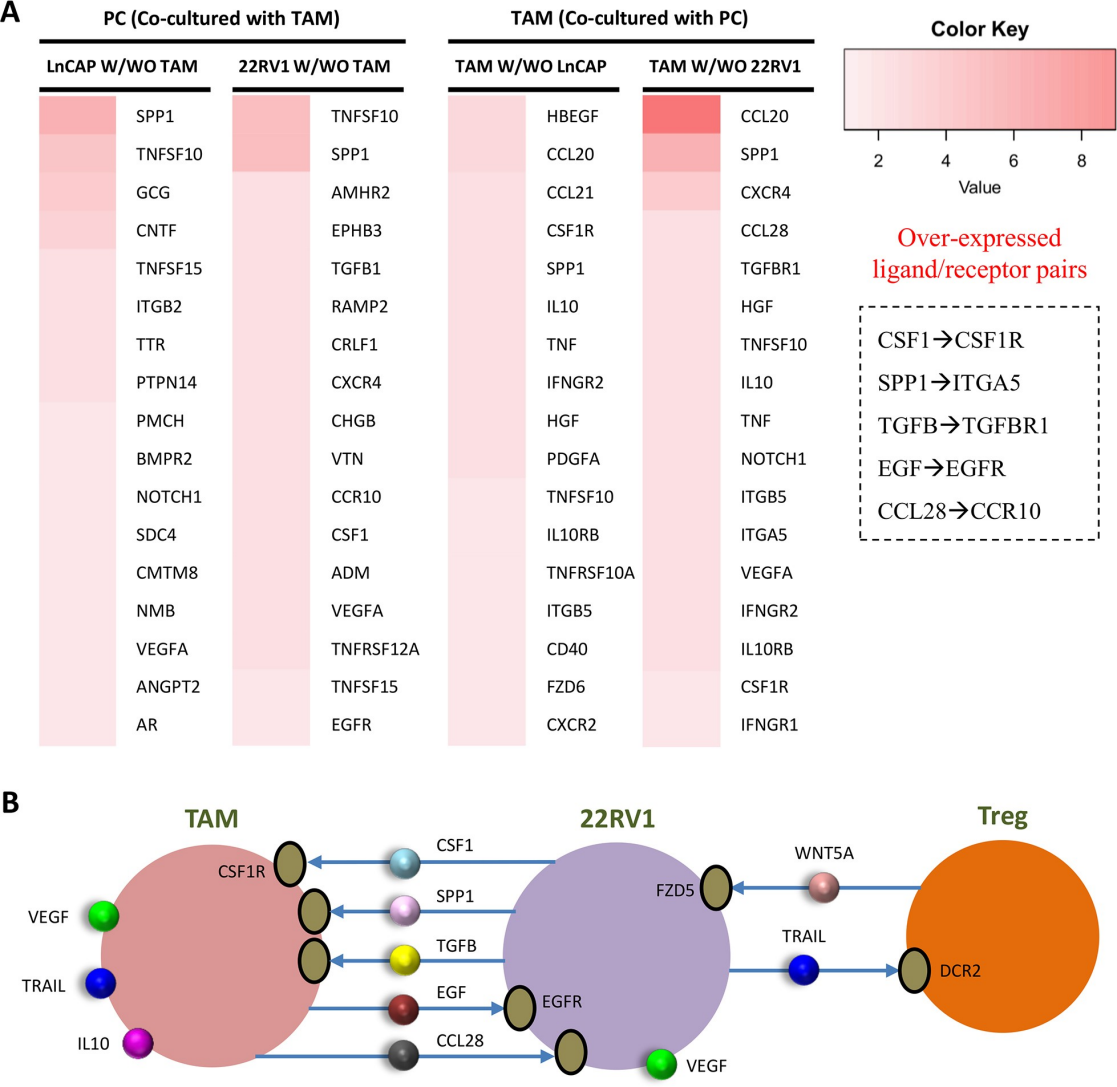
Inference of TAM-PC interactions with RNA-Seq data. (**A)** The left panel shows the RNA-seq data from the cocultured macrophage and PC LnCap and 22RV1 cells. Prostate cancer cells (LNCaP or 22RV1) were co-cultured with or without M2 macrophage (TAM) for 48 h and RNA samples were collected for RNA-seq analysis. All of the gene expression data (fold change value) were normalized with non-co-cultured counterpart cells. For example, LNCaP W/WO TAM shows the gene expression ratio of LNCaP cells co-cultured with TAM to LNCaP cells not co-cultured with TAM. The top-ranked overexpressed genes with FC>1.3 are presented. Five enriched ligand-receptor pairs were highlighted. **(B)** The inferred cell-cell interaction networks between TAM, Treg, 22RV1.

In order to validate the PC-TAM interactions inferred above, we performed coculture experiments (**Materials and Methods**). The *in vitro* experiments were designed to test the interactions of induced M2 macrophages with LNCaP cells (androgen-sensitive) or 22RV1. The RNA-seq data from the co-culture of M2 macrophages with LNCaP or 22Rv1 cells was used to validate the PC-TAM interactions. With a defined FC value > 1.3 (fold change of presence TAM to absence TAM), we totally obtained 11 over-expressed ligand genes (e.g., TNFSF10, VEGFA) and 6 receptor genes from the co-cultured LNCAP cells; and 13 ligand genes (TNFSF10, SPP1, etc.) and 12 receptor genes (e.g., EGFR) in the co-cultured 22RV1 cells. At the presence of TAMs, we found that 1) LNCaP positively expressed AR signaling axis; 2) 22RV1 secreted CSF1 and TNFSF10 (TRAIL), which potentially induced TAM recruitment and polarization, and Treg proliferation. Similarly, we obtained 27 overexpressed ligand genes (e.g., IL10) and 30 receptor genes (e.g., CSF1R) from M2 macrophages co-cultured with LNCAP cells, compared with the M2 cells without co-culture. Also, 31 ligand genes (IL10, TNFSF10, and VEGFA, etc.) and 46 receptor genes (CSF1R, TGFBR1, etc.) were over-expressed in M2 macrophage co-cultured with 22RV1 cells. **Fig 2A** shows the top-ranked overexpressed ligand and receptor genes in these three types of cells (**S1 Data**). As described in the above section, we determined the potential directional connections with high confidence scores (from iRefWeb) and obtained 5 ligand/receptor pairs between TAMs and 22RV1s (**Fig 2A**), including the positive loop PC→CSF1→TAM and TAM→EGF→PC demonstrated by other researchers [20]. Combing the above findings, **Fig 2B** revealed the cell-cell interaction network between TAM, Treg, and 22RV1. All the enriched genes corresponding to **Fig 2A** were presented in **S4 Table**.

**Fig 3:**
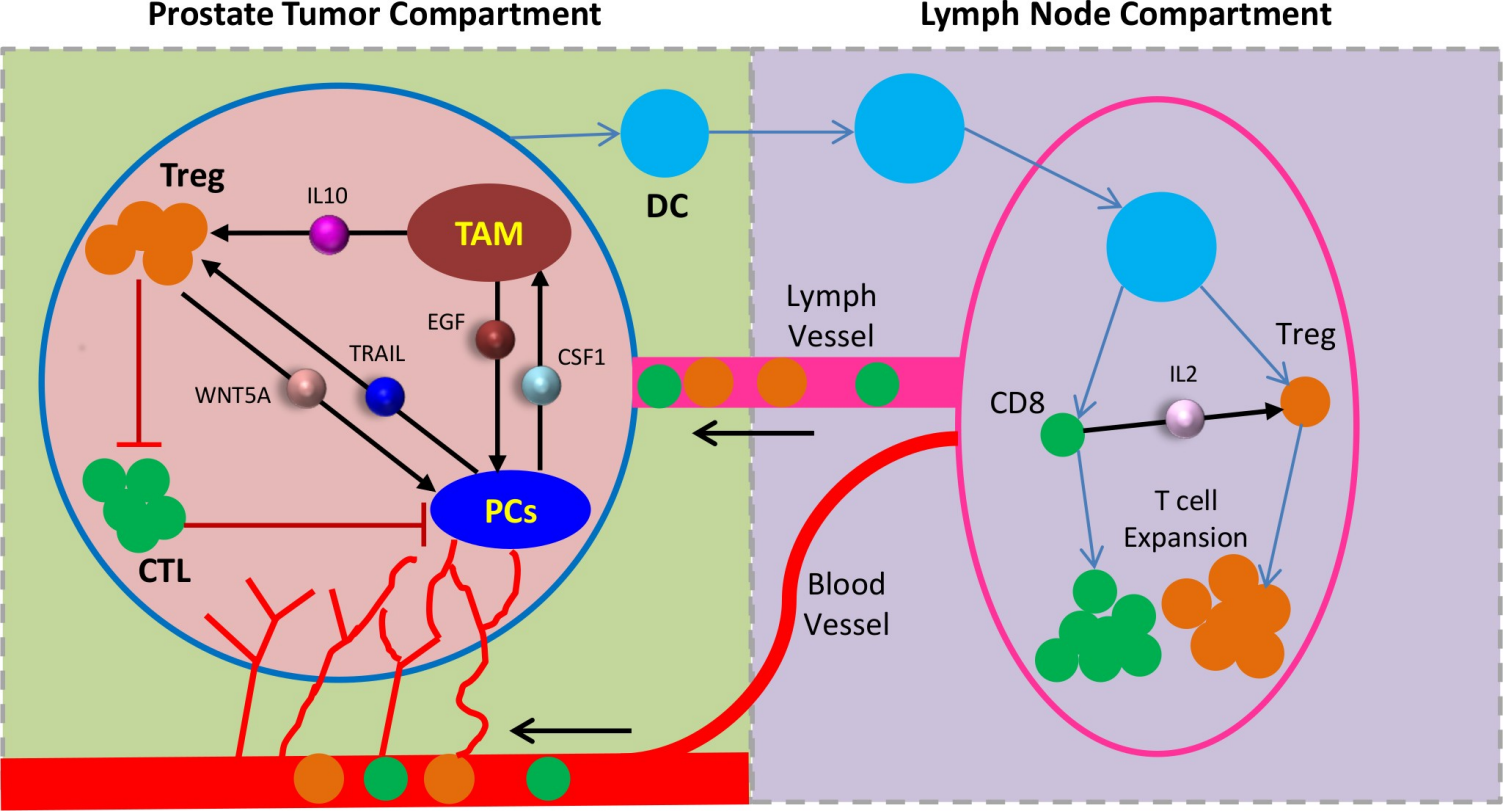
The system modeling diagram of CRPC development. The HMSM model includes two components: prostate cancer compartment (left) and lymph node compartment (right). The arrows represent cell-cell communications, which were inferred from our data or other public datasets.

Taken together, our analyses show that two potential cell-cell interaction loops appear to involve in the development of CRPC. The first loop is the secreted WNT5A from Tregs and macrophages triggers the activation of signaling pathways of cell survival and proliferation (e.g., WNT5A signaling, PI3K/AKT/AR and MAPK pathways, etc.) in androgen-resistant PCa cells. TRAIL secreted from PCs promotes Treg proliferation [32]. The second loop is ADT-induced CSF1 expression in the tumor cells stimulates TAM infiltration. Increased TAM activation leads to increased secretion of EGF and VEGF, which in turn activate AR signaling and promote angiogenesis, respectively. Combining the above information of cell-cell communications, we highlighted an integral system in the immune mE of prostate cancer that may lead to CRPC development (**Fig 3**).

### Model development

#### Hypothesis

Except the genomics data described above for the prediction of cell-cell interactions, the cell population data quantified from animal models was also needed for tumor growth modeling. Akins and Tang *et al*. developed a prostate-specific Pten^-/-^ mouse model [21, 22] and observed the dynamical changes of CD8^+^ T cells and Tregs at 2.5wk and 5wk after castration. Their studies showed that 1) castration induced an accelerated Treg expansion in mice; 2) the frequency and function of CD8^+^ T cells (CTL) was significantly increased at the early stage after castration and dropped at 5 weeks post-castration; 3) blockage of IL-2 abolished the increased expansion of Treg in lymph node following ADT. Based on the above findings, we generated the following **hypothesis** for modeling the immune mE during CRPC progression, as shown in **Fig 3**. At the early stages after castration, androgen deprivation induces apoptotic death of PCs and antigen secretion from the dying prostate tumor cells. Dendritic cells (DCs) process these antigens and present them on the cell surface. Once activated, DCs migrate to the lymph nodes and interact with T cells to initiate immune response [39, 40]. As a result, a number of activated immune cells (CD8^+^ and Treg) are quickly cloned in the prostate draining lymph nodes and infiltrated through lymph vessels. CTLs are expanded at the early stage and induced the lysis of tumor cells. However, the number of CTLs is declined at a late time after ADT due to the accelerated amplification of Tregs [22, 41]. IL-2, produced by activated CD8^+^ T cells contributes to Treg proliferation [42]. Moreover, ADT induces the PC-Treg and PC-TAM positive signaling loops, which promote the resistance of PCa and elevates the expression of key cytokines in the PCa mE [43, 44].

#### Systematically modeling the immunity leading to CRPC progression

**Fig 4:**
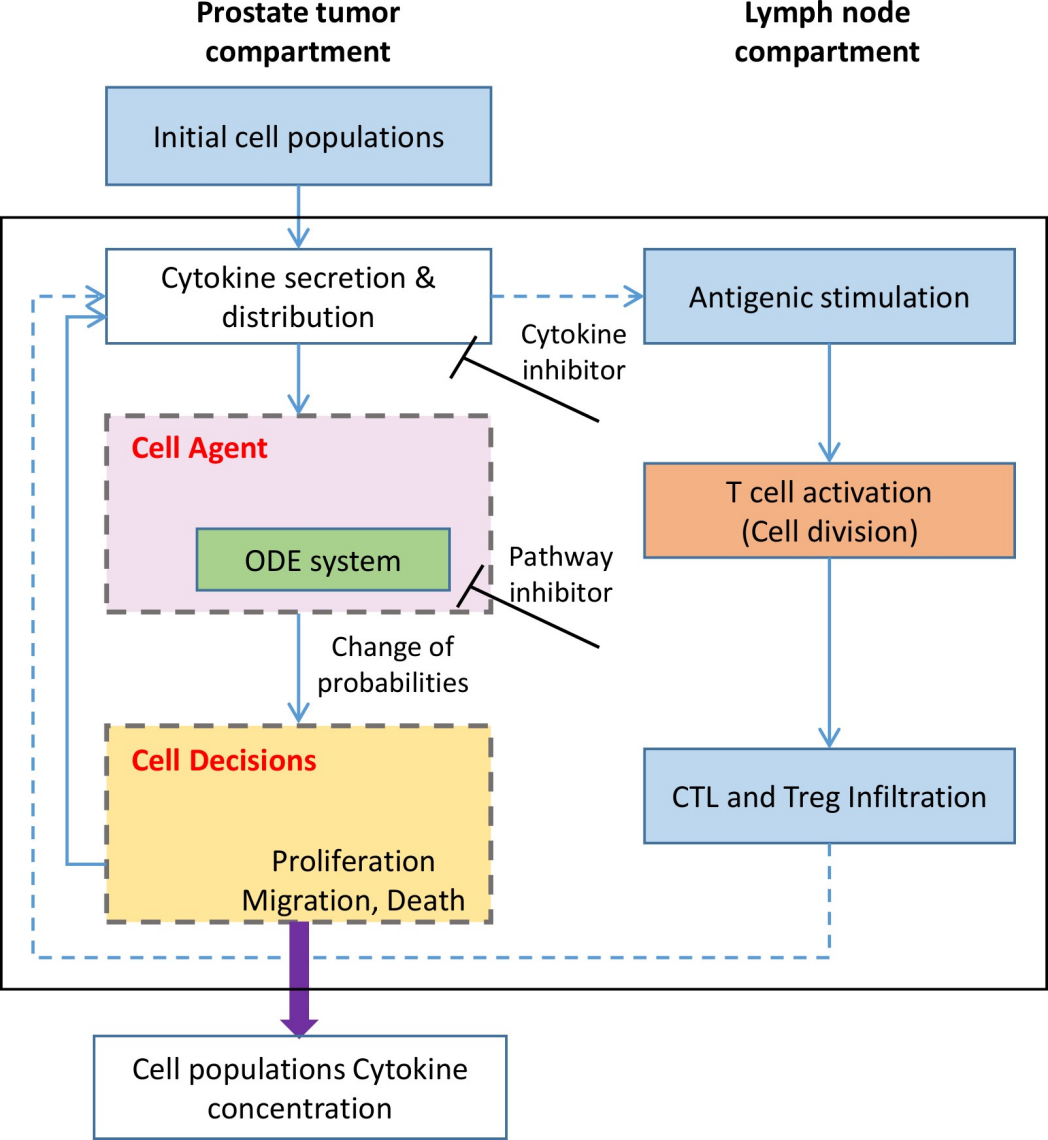
Schematic representation of computational framework of HMSM model.

To systematically understand cell-cell interactions, we established a predictive 3D **H**ybrid **M**ulti-**s**cale **M**odel (HMSM), which combines a 3D multi-scale agent-based model (ABM) for tumor growth and immune response, and an ODE system for dynamic signal transduction (**Fig 4**). Our HMSM model includes five types of cells (PC, TAM, CTL, Treg, and EC (Endothelial Cell)), which were represented by five types of cell agents of intracellular signaling events and interfaces.

**Fig 5:**
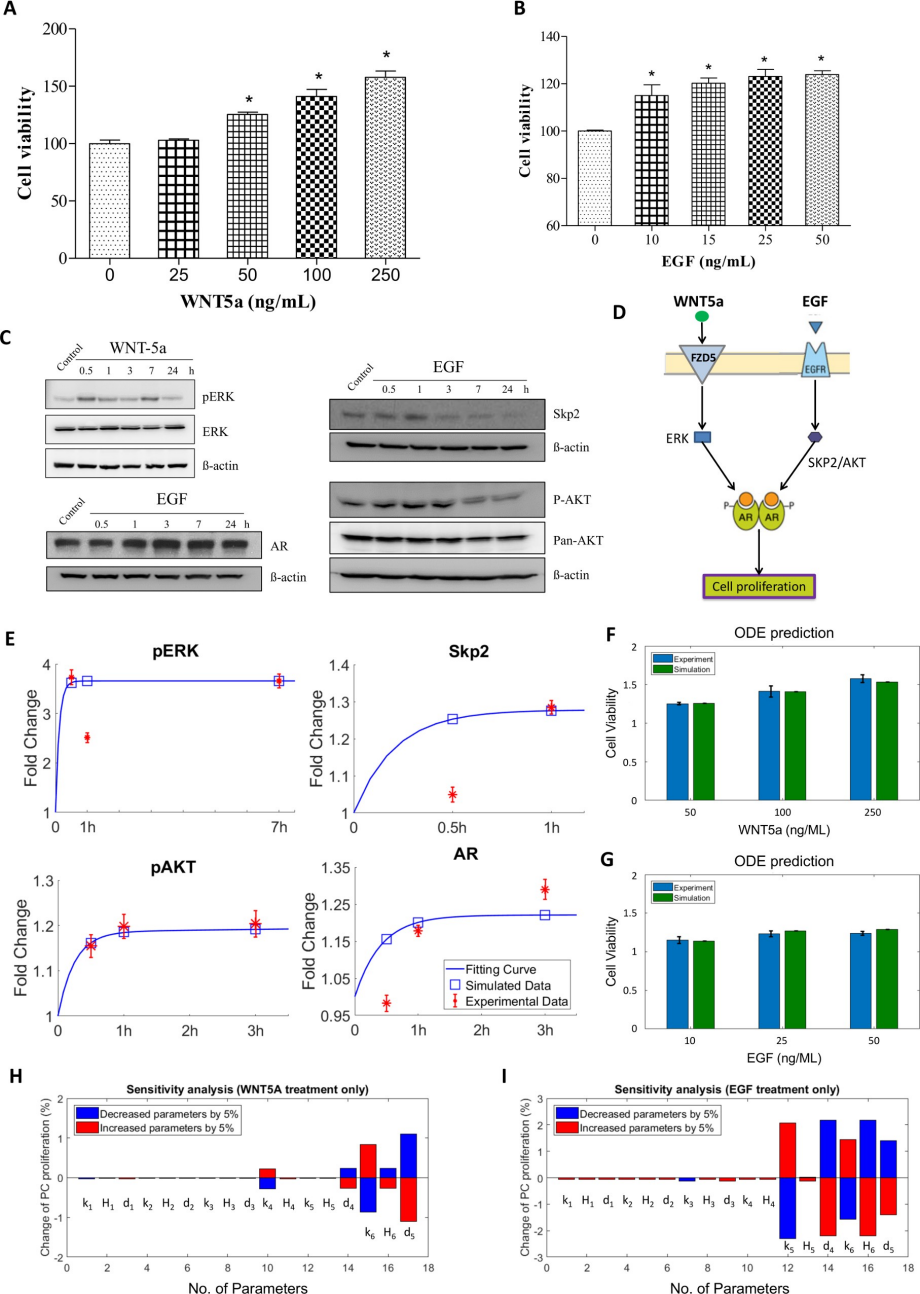
ODE modeling of WNT5A-EGF/AR signaling and experimental validation. (**A)** The effect of WNT5A on 22RV1 cell viability. (**B)** The effect of EGF on 22RV1 cell viability. (**C)** The effect of WNT5A and EGF on protein levels of Skp2, pERK, pAKT and AR. (**D)** The network topology of androgen-independent pathways in prostate cancer cells. WNT5A or EGF regulates the cellular proliferation by activating AR-related pathway. This network was represented as a series of ODE equations shown in Eq. (1–6) in the section “Materials and methods”. **(E)** The predicted values of four proteins fit the observation data well. (**F, G)** The ODE system-predicted PC proliferation at 72 hours perturbed by WNT5A (**F**) or EGF (**G**) with different doses. (**H, I)** Sensitivity analysis of the ODE system was performed under two conditions: WNT5A treatment only (**H**), and EGF treatment only (**I**). Each parameter was perturbed by increasing or decreasing 5%.

(1) PC agents represent prostate tumor cells with two possible states: androgen-dependent (sensitive), and androgen-independent (resistant). The development mechanism from androgen dependent to androgen independent PCs (CRPC) is associated with AR reactivation through AR mutations, AR variants, and gene amplifications after ADT [45, 46]. After castration, the concentration of androgen in blood and tumor tissues decreases to ~10% and 20% of the pre-treatment values, respectively [47, 48]. However, CRPC cells can synthesize dihydrotestosterone (DHT), thereby reactive AR [49]. In PC agents, we not only took into account of the androgen-independent pathway (triggered by EGF and WNT5A), but also DHT-mediated androgen-dependent signaling to determine the internal factors-associated tumor cell proliferation [46, 50]. The proliferation rates of PC cells were determined via Hill function for the androgen-dependent pathways and ODE system for the androgen-independent pathways (**S1 Text**). Therefore, we developed an ODE system to simulate WNT5A/EGF-triggered signal transduction of PC proliferation (**Fig 5**). Firstly, we observed the changes of PC proliferation treated by WNT5A or EGF with different doses (**Fig 5A and 5B**). Secondly, the key proteins associated with PC proliferation were measured using Western Blot (**Fig 5C**). We mainly used the signaling data from 0-3h for modeling in the ODE system because initial phosphorylation typically occurs around 2 hours after stimulated by extracellular ligands [51]. For Skp2, we only chose the observed values at 0.5 and 1h for model training as its expression didn’t change after 3h, compared with control. Finally, an ODE system was built on the signaling pathway network as shown in **Fig 5D**. The ODE system was described into details in “**Materials and Methods**”. All of the parameters involved in this system were estimated using GA algorithm [27, 52], and presented in the **S5 Table**. With the GA-guided optimal parameters, the ODE system fitted the experimental observations well and the ODE system stabilized after 1 hour (**Fig 5E**). In addition, **Fig 5F and 5G** indicate that the optimized ODE system recaptures the experimental observations shown in **Fig 5A and 5B**. Sensitivity analysis shows that the ODE system is quite stable when each parameter (**S5 Table**) was perturbed by a 5% increase or decrease (**Fig 5H and 5I**).

(2) TAM agents denote TAM cell population, which reside in the tumor space. TAMs are believed to promote tumor invasion and metastasis [20]. TAM infiltration is a significant unfavorable prognostic factor for prostate cancer patients [53, 54]. Our data analysis shows that the direct interactions between TAMs and PCs appear to be one of the main reasons for the resistance of PCs to androgen deprivation.

(3) CTL agents represent the CD8^+^ effector T cells, which mediate tumor cell lysis through cell-cell contact. Antigen-presenting DCs migrate to the draining lymph nodes and present the antigen to T cells, so that the CD4^+^ T helper and CD8^+^ effector cells are activated [55, 56]. After undergoing up to 8 generations of division, these T cells acquire effector functions, such as cytotoxicity [57]. Activated CTLs migrate to the tumor through lymph vessels or peripheral blood and contribute to tumor cell death either by releasing perforins that create holes in the target cell’s membrane or by triggering apoptosis in the target cells.

(4) Treg agents represent the immune-suppressive T cell population responsible for the maintenance of peripheral tolerance and have been implicated in the suppression of tumor immunity. Tregs have been shown to inhibit tumor-specific T cell functions, such as the cytotoxic effects of CTLs [58]. Tregs require ligand-specific activation and cell-cell communication to exert their suppressive activities [59]. The factor TRAIL, expressed in PC, can promote the growth of Tregs [32]. Also, the secreted IL-2 by CTLs promoted Treg expansion [42, 60]. In our HMSM model, Tregs in the tumor space was initially activated in lymph node and then infiltrated to the tumor to inhibit/prevent the cytotoxic effects of CTLs by suppressing CTL proliferation (**Materials and Methods**).

(5) EC agent is defined to simulate tumor angiogenesis in prostate cancer mE. After ADT, TAMs and PCs secret VEGF to induce new vessels to sprout from the pre-existing vasculature towards the center of a tumor, providing nutrients to the starving tumor cells and thereby stimulating tumor growth. VEGF spreads to the surrounding tumor tissues and is also consumed by endothelial cells [61]. The motion (branching or proliferation) of individual ECs located at the tip of a capillary sprout governs the movement of the whole sprout, and chemotaxis in response to VEGF gradients [62].

#### 3D Multi-scale modeling

As shown in **Fig 3**, CRPC progression is simulated at intracellular, intercellular, and tissue levels in the HMSM model. Intracellular signal transduction was modeled by Hill functions or ODEs to represent the rates of proliferation and apoptosis (**S1 Text**).

The intercellular communication in HMSM reflects the relationship between cancer cells, immune cells and tumor mE evolution during CRPC progression through the following aspects. 1) ADT induces the prostate tumor cells to express CSF1, which promotes macrophage activation and infiltration [20]. 2) Enhanced infiltration of TAM results in the accumulation of VEGF, IL10, and EGF, promoting angiogenesis, tumor growth, and immune suppression [20, 38]. 3) The positive feedback loop between Tregs and PCs leads to PC proliferation stimulated by WNT5A and Treg growth induced by TRAIL [32]. 4) The production of IL-2 from CD8^+^ promotes Treg proliferation [63]. 5) CTLs recognize tumor cells in their local regions and migrate toward these target cells for clearance [64]. 6) Accumulation of Tregs potentially inhibits the proliferation of CTLs, resulting in the suppression of anti-tumor immune responses [58].

The tissue scale reflects 3D prostate tumor growth and cell responses to castration via various intercellular cell-cell interactions spatially and temporally as described above. At the tissue level, intracellular signaling pathways were triggered by the secreted cytokines, such as WNT5A, TRAIL, CSF1, EGF, IL-2, VEGF, and IL10, locally via the interfaces of cell agents, and result in the changes in the cells’ fate and behaviors, which in turn modulate the tumor mE for cell growth and response to treatment. In this scale, we defined the dynamic 3D distribution of the key cytokines secreted from four types of cells. VEGF can be secreted from both PCs and TAMs, leading to the initiation of tumor angiogenesis. In addition, the distribution of cell populations in tumor mE is defined by cell proliferation and migration in 3D ECM. In particularly, we defined the immune cell infiltration from lymph nodes to the tumor bed via lymph vessels.

#### Model implementation

At the beginning of simulation, 200, 100, 2, and 2 of PC, TAM, CD8^+^ T, and Treg cells were used, respectively, to mimic the initial stage for prostate tumor cells spreading to a new location. Based on all the observed data mentioned above, we manually tuned all the parameters associated with ABM part in HMSM for optimizing the data fitting (see the details in **S1.9 Text**). All the parameters of ABM model are presented in **S6 Table**. After optimization, we selected WNT5A antagonist, CSF1R inhibitor PLX3397 (PLX) [20], EGFR inhibitor, anti-IL2 mAb [22] as the representatives of agents, respectively, in HMSM to predict the therapeutic effects *in silico*. The number of each cell type in HMSM was recorded every 2 hours [25, 52, 65]. The drug effects were represented as the fold changes in the number of tumor and immune cells following treatments. Our HMSM model simulated a series of biological events up to 9 weeks, including prostate tumor growth, ADT-induced immune response, and the emergence of CRPC (**S4 Fig**).

**Fig 6:**
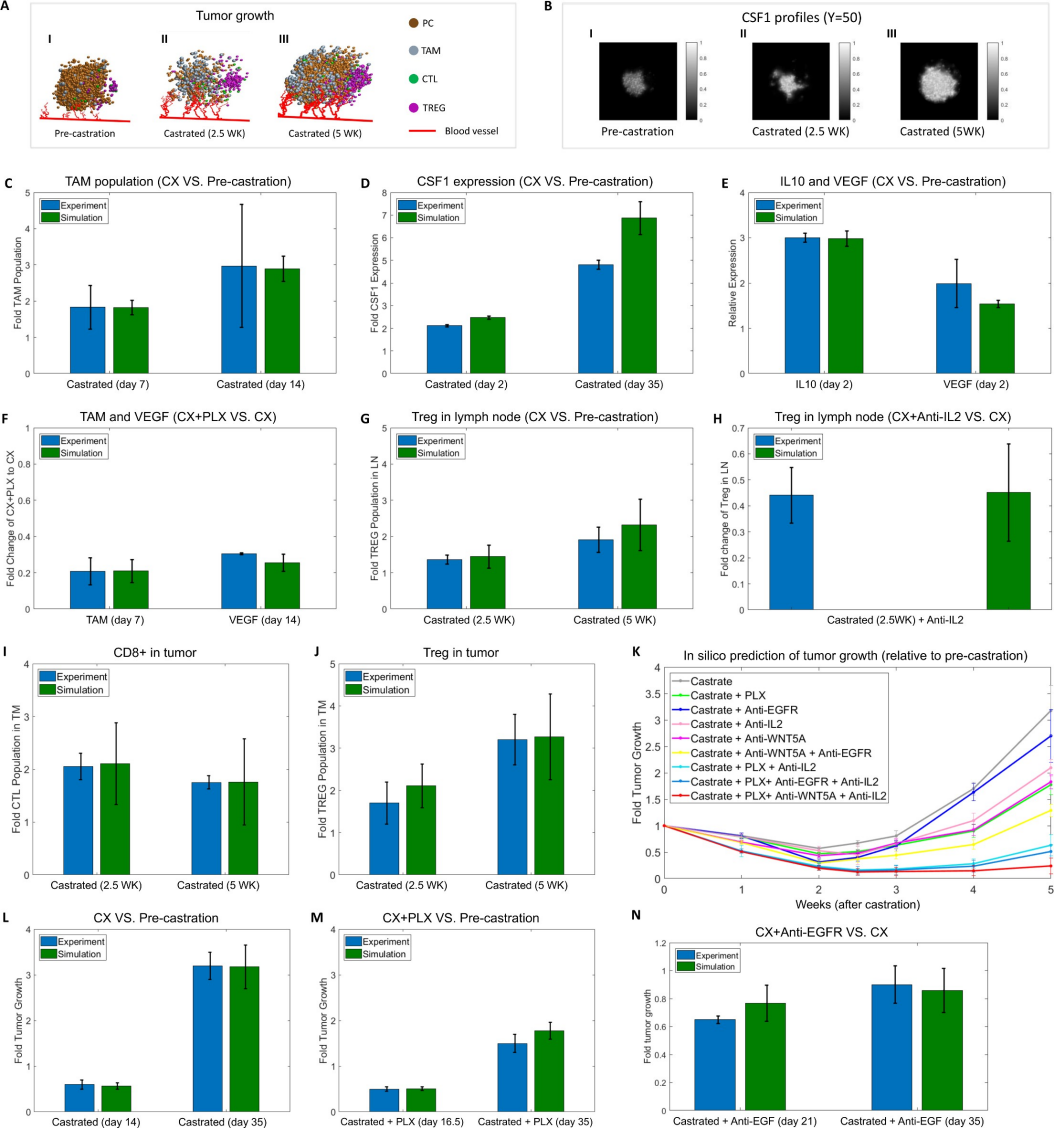
Experimental observation and *in silico* prediction of HMSM Model. The bars with blue color are experimental-measured values, and the bars with green color are predicted values in the HMSM model. CX: Castration. Time 0 denotes pre-castration. (**A)** A simulation example of prostate tumor before/after castration. I) pre-castration; II) 2.5 weeks after castration; III) 5 weeks after castration. **(B)** A simulation example of cytokine profiles before/after castration. I) pre-castration; II) 2.5 weeks after castration; III) 5 weeks after castration. The slices are extracted when Y = 50. Y is the Y axis. **(C)** TAMs are elevated by ADT in prostate cancer (day 7 and day 14 castration). **(D)** CSF1 protein level was analyzed from castrated mice (day 2 and day 35 castration). **(E)** Relative gene expression of IL10 and VEGF at 48hours after ADT. **(F)** PLX lowered macrophage levels and VEGF expression after ADT. **(G)** The number of Treg cells is increased in lymph nodes at 2.5 weeks and 5 weeks post-castration. **(H)** Treg expansion in lymph nodes was reduced by IL-2 neutralization. **(I)** *In silico* prediction of CD8^+^ cells in the castrated tumor at 2.5 and 5 weeks. **(J)** *In silico* prediction of Treg cells in the castrated tumor at 2.5 and 5 weeks. **(K)** *In silico* prediction of single or combined treatment on PC growth after castration relative to pre-castration. **(L-N)** The predictions and experimental validations for prostate tumor growth with castration only (**L**) or plus CSF1R (**M**) or plus EGFR inhibition (**N**) after castration.

A simulation example of prostate tumor growth before and after castration in the immune suppressed mE is shown in **Fig 6A**. Panel I represents a simulated PC tumor status before castration. CD8^+^ T cells and Tregs gradually infiltrated to the tumor space from lymph nodes following the activation induced by prostate-specific antigen-presenting DCs. Castration leads to a rapid decrease of the androgen concentration and increase of immune infiltration, resulting in tumor regression. Panel II shows that most of the tumor cells are cleared at 2.5 wks after castration. After that, CRPC phase eventually emerges, manifested as the elevated TAM infiltration and increased Treg expansion, which appears to contribute to the tumor relapse and immune suppression in the prostate mE (Panel III). **Fig 6B** presents the dynamics of CSF1 profiles associated with CRPC development (**S5–S7 Figs**). CSF1 accumulation is transitorily decreased in the early stage after castration and rapidly rebounded as CRPC occurrence.

### Model evaluation

To test the fit of our HMSM model to the training data, *in silico* simulations under several contexts were evaluated using the experimental data from our laboratory, as well as the data from previously reported studies (**S2 Data**). Firstly, we simulated the whole process of prostate tumor growth from the initial state to 6 wks after castration. The dynamic changes of TAM population, CSF1 expression, and TAM-secreted protumorigenic cytokines (e.g., IL10, and VEGF, etc.) in the simulated mE were predicted. **Fig 6C** shows that TAM population are increased by 1.826±0.2 folds at day 7 and continued to increase to 2.891±0.353 folds at the day 14 after castration. **Fig 6D** represents the simulated expression of CSF1 from prostate cancer cells. The expression of CSF1 in PCs is significantly increased after ADT, which is close to the measured results from the subcutaneous mouse model [20]. Moreover, the predicted expressions of IL10 and VEGF in TAMs have increased 2.98±0.171 and 1.54±0.078 folds at two days post-castration (**Fig 6E**). We also predicted the effect of the CSF1R inhibitor on the distribution of the cell population after castration. As shown in **Fig 6F**, inhibition of CSF1R with PLX after ADT results in a 5-fold reduction of the TAM population in the early stage, compared with the results in castration only. The CSF1R blockade appears to inhibit macrophage proliferation, and lower TAM-induced expression of VEGF, which potentially delays the emergence of CRPC. The simulated results shown in **Fig 6C–6F** are consistent with the experimental data reported in [20].

To examine the dynamical changes of immune responses, we calculated the changes of the Treg population after castration in the simulated system. As shown in **Fig 6G**, the number of Treg cells is significantly increased in lymph nodes at 2.5 weeks and 5 weeks post-castration compared to that from pre-castration. This simulation result is close to the previous findings reported by Tang *et al*. [22]. In addition, **Fig 6H** shows that Treg expansion is prevented by IL2 neutralization, suggesting the increased IL2 after castration and immunization promotes Treg expansion [22]. Taken together, we found that the HMSM model fits the observed data very well under different contexts.

### Model validation

To further validate the reliability of our HMSM model optimized above, we compared the simulation results with additional experimental data (**S2 Data**) generated from Pten^-/-^ prostate cancer mouse model [21, 27]. Fourteen weeks-old mice were castrated, and the relative changes of immune cells (Treg and CD8^+^ T cells) in tumor space were observed at the 2.5 weeks and 5 weeks after castration [21]. **Fig 6I and 6J** show that castration induces infiltration of Treg cells into the tumor area in the prostate tumor-bearing mice. However, the accumulation of functional CD8^+^ T cells in the prostate tumor is not long-lasting, evident at 2.5 weeks after castration but reduced at 5 weeks after castration. The measured CD8^+^ T cells at 2.5 weeks and 5 weeks after castration are around 2.05±0.25 and 1.75±0.125 folds of pre-castration, respectively [27]. **Fig 6I** indicates that the prediction of the CD8^+^ population (2.107±0.775, and 1.7606±0.8141 folds) in the HMSM model is consistent with the experimental observations. Moreover, the number of Tregs was significantly increased to 1.7±0.5 and 3.2±0.6 folds in the tumors at 2.5 and 5 weeks after castration, respectively [27]. HMSM model simulation shows that the predicted changes of the Treg population at 2.5 and 5 weeks after castration, and our predicted results are close to the experimental observations (**Fig 6J**). In summary, our testing experimental data further confirms that the outputs of HMSM model are reliable.

### Prediction and validation of therapeutic outcomes

To identify the potential therapeutic targets of PCa in the immunosuppressive prostate cancer mE, we predicted the effects of single or combined treatments with castration on PCa growth using the established HMSM model (**S2 Data**). Our experimental data revealed WNT5A was a potential factor associated with CRPC development. Therefore, we simulated the effect of WNT5A neutralization on PC growth with our *in silico* model. Recent studies indicate that CSF1R inhibitor (PLX3397) [20, 66] and IL-2 neutralization [22] revealed the effects for immune re-activation after ADT. In addition, early studies have reported the efficacy of EGFR inhibitors (e.g. erlotinib, canertinib, and cetuximab, etc.) in castration-resistant prostate cancer *in vitro* and *in vivo*, and claimed that EGFR inhibition might improve the outcome of patients with CRPC [28, 67, 68]. Therefore, we mainly tested the anti-tumor effects of four representative agents in HMSM, including anti-WNT5A antibody, PLX3997, Anti-IL-2 antibody (IL-2 neutralization), and EGFR inhibitor.

**Fig 6K** shows the predicted outcomes from single or combined treatments relative to pre-castration. Prostate tumor cells were reduced sharply at the first 2 weeks after castration and then re-expanded continuously (silver curve). The combined treatment with castration and a single agent (Anti-WNT5A, PLX, Anti-IL-2, or EGFR inhibitor) yields a better treatment response than that from the castration only group. Comparing with Anti-IL-2 and EGFR inhibitor, a combination of castration with Anti-WNT5A or PLX yields better anti-tumor responses, indicating that blockade of the PC-Treg or PC-TAM interaction may effectively reduce tumor cell growth. In addition, the poor response was observed in the combined treatment group with castration plus EGFR inhibitor, compared with the other combined treatment groups. The optimal prediction outcome was achieved from the treatment group with a combination of PLX, Anti-WNT5A, and Anti-IL-2 (red curve) after castration, revealing that the activation of both Treg and TAM appears to contribute to CRPC development. Moreover, we compared the predicted results with the experimental observations reported previously. **Fig 6L** shows that the tumor growth rebounded approximately 3 times after castration, paralleling the emergence of CRPC observed in the clinical setting [20]. The addition of CSF1R inhibitor PLX3997 to castration resulted in a significant delay in the onset of CRPC (**Fig 6M**) [20]. Also, Anti-EGFR leads to 0.77±0.128 and 0.86±0.157 fold tumor growth at 3 and 5 weeks after castration relative to castration only (**Fig 6N**), which are close to the experimental observation of *in vivo* effects of EGFR inhibitors in 22RV1 xenografts mice model [28]. Above analyses indicate that the predictive capabilities of our HMSM model are high and the model-based predictions are reliable.

## Discussion

The focus of this work is to explore CRPC progression in the immune mE and to develop optimal treatment strategies *in silico* to improve therapeutic responses of CRPC. To systematically understand the role of the immune system in CRPC development, we generated RNA-seq data and integrated it with the GEO datasets. Through the analysis of these data, we found the potential factors/cytokines (e.g., WNT5A, and TRAIL) associated with PC-immune interactions. Elevated levels of WNT5A have been reported in melanomas, lung cancer, breast cancer, and gastric cancer [69–73]. Lee, *et al*. investigated the Cap-bone stromal cells interaction, and reported that WNT5A secreted by bone stromal cells increases BMP-6 expression in Cap, thereby leading to Cap cell proliferation [33]. Our study demonstrated that WNT5A induced the activation of androgen-independent pathways and the elevated expression of TRAIL in CRPC cells after castration, indicating the enhancement of PC growth and immune suppression. As a type 2 membrane protein belonging to TNF superfamily, TRAIL is known to play a pivotal role in the immune regulation and antitumor immunity [74–76]. Early studies revealed that TRAIL has the potential to promote Treg proliferation in certain situations [77]. Ikeda and coworkers demonstrated that the proliferative effect of TRAIL on Tregs becomes apparent in autoimmunity [32]. The exploration of TRAIL function in prostate cancer may be of considerable significance for understanding CRPC mechanisms.

We are the first to systemically model the CRPC development in the immune mE using an integrated 3D system (**S8 Fig**). In our HMSM model, we simulated the PC growth before and after castration. The first stage covers a sequence of key biological events, including DC maturation, T cell activation, and division in lymph nodes triggered by DC, T cell migration and infiltration. The second stage denotes the initial castration therapy (5 weeks), in which the AR signaling reactivation appears around 2 weeks after castration (**S4 Fig**). Therefore, the proposed model provides a new way to present the dynamic changes in tumor growth, immune response, and drug treatment effect.

We also provide a novel computational platform to optimize the potential target therapy on the castrated PCs. ADT is a standard treatment for PC patients, including surgical castration, and AR disruption with pharmacologic interventions (such as MDV3100 (enzalutamide) [78]). However, clinical studies indicate that AR antagonist can induce AR T878A mutation and result in AR reactivation [79–81]. Our analysis of a representative GEO dataset (GSE67980) [82] also revealed that AR expression was increased when the patients with CRPC treated by enzalutamide (**S9 Fig**). In recent years, active immunotherapy, such as therapeutic vaccines, provide new strategies for overcoming tumor-mediated immune suppression [83]. Multifaceted approaches that combine vaccine with targeted therapies may have the potential to improve the current therapeutic outcomes by targeting the suppressive immune microenvironment and tumor survival. In the present study, we evaluated several new therapeutic strategies *in silico* with our optimized HMSM model. The simulated results showed that the optimal prediction outcome was achieved from the treatment group with a combination of PLX, Anti-WNT5A, and Anti-IL-2 after castration, revealing the important role of Treg and TAM activation.

Moreover, the proposed model includes a large number of parameters, and most of the parameters were tuned manually or determined based on the experimental results. In order to confirm the variability of the simulated results from the developed 3D hybrid multi-scale model, a parameter sensitivity analysis was performed by measuring the impact of a small perturbation (5% increase) of individual 34 key parameters on the prostate tumor cell populations (5wk after castration). We found that 1^st^ and 2^nd^ parameters (the basic proliferation rates caused by castration-dependent and castration-independent pathways in PCs) were more sensitive than others (**S10 Fig**). It indicates that ADT induced prostate cancer cells to progress and further express cytokines to promote CRPC occurence. The sensitivity analysis showed the changes in model outcomes were under 4%, indicating that the outcomes of the optimized model were stable. We also tested the effect of initial cell numbers and cut-off values in the ABM rules on the model variability. **S11 Fig** and **S12 Fig** show that simulated tumor growth is not sensitive to the perturbations on the initialization of cell number and the cut-off values in the ABM rules. We did additional analysis with the experimental time points overlaid as dots at the observed times, our results indicate that the optimized HMSM model is reliable (**S13 Fig**).

Although a number of mathematical approaches have been introduced to model the tumor growth and drug resistance in recent years, most of the well-defined 3D agent-based models not only neglect the stage-structured immune response during the tumor initialization and development, but also did not simulate the dynamics of intracellular pathways in the cell-cell communications [25, 65]. Solovyev *et al*. was the first to put forward the concept “hybrid model”, which combined ODE model and agent-based model to mimic signal transduction processes at the intracellular scale, stochastic cell behaviors at the intercellular scale, and the dynamic distribution of growth factors at the tissue scale [84]. However, their model was only designed for two-dimensional space so that it cannot be used in 3D tumor study. Our 3D Hybrid model (HMSM) overcomes the limitations of existing models described above, and creates a new paradigm for systematically understanding the immunity leading to CRPC.

There are several limitations of our HMSM model. We used some experimental data from *in vitro* 2D culture to model 3D microenvironment in this study. Ideally, experimental data obtained directly from 3D tissues can better reflect actual environmental status. However, such types of data are not easily available due to animal study settings. Moreover, using limited available animal data for ABM model training, validation, and prediction may not be enough for the validation of our large-scale-based ABM model. Incorporating much more observed data will increase the reliability of the model outcome. In the future, we will collect tumor tissue data from patients with PCa before and after castration to verify our 3D model. We will develop heterogeneity scoring approaches to evaluate cell-level heterogeneity (receptor expression) and tumor-level heterogeneity (cytokine levels, and geometry). We will extend our model to simulate the effect of new blood vessels on the tumor growth, e.g. modeling increased cancer cell migration and invasion. To better address clinically relevant issues, we will further improve our model in terms of varying-degree inhibition with inhibitors, enabling it to predict dose-related treatment outcomes.

## Materials and methods

### Experiment

#### 1) Cell culture

LNCaP and THP1 cells were purchased from ATCC (Manassas, VA) and 22RV1 cells were a gift from Dr. Lin’s lab in Wake Forest Medical Center. LNCaP cells were maintained in RPMI-1640 medium supplemented with 10% bovine calf serum (FBS), 2 mM L-glutamine, 10 mM HEPES, 1 mM sodium pyruvate, 4500 mg/L glucose, 100 IU/mL penicillin, 100 μg/mL streptomycin and 1500 mg/L sodium bicarbonate, 22RV1 cells were maintained in DMEM medium containing 10% FBS, 2 mM L-glutamine, 100 IU/mL penicillin and 100 μg/mL streptomycin. THP1 cells were cultured with RPMI-1640 medium supplemented with 10% FBS, 2 mM L-glutamine 0.05 mM 2-mercaptoethanol, 100 IU/mL penicillin and 100 μg/mL streptomycin.

#### 2) Cell viability analysis for the 22RV1 cells treated with EGF

2 X 10^4^ 22RV1 cells were plated in 24-well plates and incubated overnight. The cells were then treated w/wo EGF (1, 5, 10, 20, 50 ng/mL) for 72 hours. Cell viability was determined using a modified thiazolyl blue tetrazolium bromide (MTT) (Acros Organics, Thermo Fisher Scientific, New Jersey) method as described previously [85]. Briefly, 125 μL of 5 mg/mL MTT reagent in PBS was added to each well and incubated for 4 hours in a CO2 incubator. Cells were then lysed by adding 50 μL lysis buffer (20% SDS, 50% N, N, N-dimethyl formamide (DMF), PH4.7) at 37°C. The absorbance at 560 nm was measured using a spectrophotometer (Molecular Devices, Sunnyvale, CA).

#### 3) Coculture of prostate cancer cells and macrophages

2 X 10^5^ THP1 monocytes were seeded onto 6 well plates overnight and then treated with 10 ng/ml of 12-O-tetradecanoylphorbol-I3-acetate (PMA) for 24 h. The PMA containing medium was replaced with serum-free medium and culture for another 2 days. For the M2 cell differentiation, the cell was treated with 25 ng/ml of IL4 and 25 ng/ml of IL13 for 48 h. The differentiated cells were washed 3 times with PBS and 4 ml of RPMI medium containing 10% FBS, 2 mM L-glutamine, 100 IU/mL penicillin and 100 μg/mL streptomycin was added into each well. Two days prior to the co-culture experiment, 2 X 10^5^ of LnCap and 22RV1 cells were seeded into 0.4 μM transwell inserts, respectively. For coculture, the culture medium in the inserts with prostate cells was removed and transferred onto the top of the 6-well plates with differentiated TPH1 cells. 2 mL of RPMI medium was added into each insert. Prostate cancer cells and M2 macrophage were co-cultured for an additional 48 hours, and cells were harvested for RNA analysis.

#### 4) Treatment of 22RV1 cells with human recombinant WNT5A protein

The human recombinant WNT-5A protein was obtained from R&D System. 22RV1 cells were cultured in the 6-well plates. 80% confluence cells were incubated with serum-free medium for 24 h and then treated with 250ng/mL WNT5A for up to 24 h. The cells were then harvested for RNA and protein analysis using Western blot and quantitative real-time PCR.

#### 5) Western blotting

The differentiated THP1 cells were lysed with 1 X RIPA buffer supplemented with protease and phosphatase inhibitor cocktail (Roche Applied Science, Indianapolis, IN) and stored in aliquots at -20°C until use. Twenty micrograms of cell lysates were denatured by boiling, and separated by SDS-PAGE. The separated proteins were then transferred to a nitrocellulose membrane (BioRad). The membranes were blocked using 5% non-fat dry milk for 1 h at room temperature and probed with antibodies overnight. After incubated with IgG horseradish peroxidase-conjugated secondary antibodies (Cell Signaling, Beverly, MA) for 2 h at room temperature, the immunoblots were developed using the enhanced chemiluminescence (ECL) reagent (Cell Signaling, Beverly, MA) and visualized using a FluroChemQ processor (Proteinsimple, Santa Clara, CA). The antibodies used in this study include CD206, ERK, pERK. AKT, pAKT, foxo1, Glut1, N-cad and skp2, which were obtained from Cell Signaling Technologies.

#### 6) Quantitative Real-time PCR (qRT-PCR)

The qPCR amplification was performed in a 20uL reaction mixture containing 100 ng of cDNA, 10 uL 2 X All-in-OneTM qPCR mix (GeneCopoeia, Rockville, MD), 0.3 mM of upstream and downstream primers and nuclear-free water. The PCR reaction was conducted with 1 cycle at 95°C for 10 min, 40 cycles at 95°C for 15 s, 40°C for 30 s and 60°C for 1 min, followed by dissociation curve analysis distinguishing PCR products. The expression level of a gene was normalized with the endogenous control gene β-actin. The relative changes of genes were calculated using the 2-ΔΔCT method and presented as mean ± SD (n = 3). The sequences of the paired sense and antisense primers for human Antigen receptor, TNF-10, FTZ1, and β-actin are listed in the **S7 Table**.

#### 7) RNA isolation and sequencing

Total RNA was extracted from the cells using an RNeasy Mini kit (Qiagen, Valencia, CA) according to the manufacturer's instructions. The quality and quantity of total RNA were verified spectrophotometrically (NanoDrop 1000 spectrometer; Thermo Scientific, Wilmington, DE, USA) and electrophoretically (Bioanalyzer 2100; Agilent Technologies, Palo Alto, CA, USA). The mRNA libraries were prepared according to the TruSeq RNA Sample Prep Kit protocol (Illumina, San Diego, CA, USA) and sequenced using Illumina HiSeq2000 DNA sequence analyzer. RNA-seq reads were aligned with the reference genome using TopHat [86]. The resulting alignment files were input to Cufflinks to generate transcriptome assemblies, and the Fragments per Kilobase of Transcript per Million fragments mapped (FPKM) values of isoforms were calculated for individual genes [87].

### ODE-based modeling of EGF-driven Androgen independent pathway

Androgen Receptor (AR) not only can be activated by extracellular growth factor through androgen-independent pathway network, but also by DHT from the tumor microenvironment through androgen dependent pathway. The activation or inhibition of these two ways depends on the stages of CRPC progression. In our study, we developed an ODE model to simulate the effect of WNT5A/EGF-triggered androgen-independent pathway on the proliferation of prostate cancer cells (**Fig 5D**). The extracellular concentration of WNT5A and EGF are the input variables for the ODEs. In the training of ODEs, the input parameters (WNT5A or EGF) were restricted to the range [0, 1], and the maximal value “1” represents the maximal dose of ligands we used in our experiment. The output is the fold change of proliferation rates relative to no stimulation. The ODE system has the following form:

$$\frac{d[ERK]}{dt}=k_{1}\frac{\left[WNT5A\right]}{H_{1}+[WNT5A]}-d_{1}[ERK]$$ (1)

$$\frac{d[Skp2]}{dt}=k_{2}\frac{\left[EGF\right]}{H_{2}+[EGF]}-d_{2}[Skp2]$$ (2)

$$\frac{d[AKT]}{dt}=k_{3}\frac{\left[Skp2\right]}{H_{3}+[Skp2]}-d_{3}[AKT]$$ (3)

$$\frac{d[AR]}{dt}={(1-D1)*k}_{4}\frac{\left[ERK\right]}{H_{4}+[ERK]}+(1-D2)*k_{5}\frac{\left[AKT\right]}{H_{5}+[AKT]}-d_{4}[AR]$$ (4)

$$\frac{d[Prol]}{dt}=k_{6}\frac{\left[AR\right]}{H_{6}+[AR]}-d_{5}[prol]$$ (5)

As mentioned above, our phosphor-proteomics data covered the key signaling proteins (pERK, pAKT, AR, and Skp2), which were involved in this androgen-independent signaling network (**Fig 5C**). The effect of WNT5A and EGF on 22RV1 cell proliferation were also presented in **Fig 5A and 5B**. All above parameters involved in this ODE system were estimated by optimizing formula (6) via the GA algorithm [27]:

$$\theta^{*}=argmin\;\sum_{i\epsilon I1,t\epsilon T1}\left|X_{i}^{t}-{\hat{X}}_{i}^{t}(\theta )\right|$$ (6)

Where $X_{i}^{t}$ and ${\hat{X}}_{i}^{t}(\theta )$ denote the measurement from the experiments and the theoretical results obtained from the ODE model of protein *i* at the time point *t*. The parameter vector *θ* = {*k*_1_,*H*_1_,*d*_1_,……,*k*_6_,*H*_5_,*d*_5_} in above formulas (1–5) can be obtained by formula (6). D1 and D2 represent the inhibitors of WNT5A and EGF pathways, respectively. The set *I*1 is the indexes of observed proteins in this signaling network, and time series set *T*1 = {0, 30min, 60min, 420min} covers all the time points related with experimental data (**Fig 5C**). **S5 Table** represents the estimated values of all parameters. The fitting accuracy of the predicted and measured values of key proteins is shown in **Fig 5**.

### The agent-based model of CRPC progression

We defined five types of agents in the ABM model to represent PC, TAM, CTL, Treg, and EC, respectively (**Fig 3**). The ABM model simulates the effects of various cell-cell interactions on prostate tumor growth, angiogenesis, programmed immune response, and drug response in a simulated mE. We initialized the simulated microenvironment as a cuboid, which consists of two connected cubes. One is for the growth and proliferation of mixed PC, TAM, CTL, Treg, and EC compartments (tumor space) and the other is for the activation and division of T cells triggered by matured DC in lymph node and T cell infiltration from its lymph vessels. The proposed model simulated a series of key biological events involved in tumor growth, immune response, and CRPC development (**S4 Fig**). The details of time points related with these events were described in the **S1 Text**.

This multi-scale modeling includes intracellular, intercellular and tissue scales, which are illustrated in the **S8 Fig**, and described into details in the following sections. Detailed flowcharts of each agent were illustrated in the **S1 Text**. Individual cell behaviors were simulated by probability-based rule implementation [52, 65]. A cell senses the hints in its neighborhood such as local cytokines and drugs and adjusts itself with the embedded signaling pathways, and outputs the corresponding changes on its cell behaviors, including proliferation, survival, differentiation, migration, and cytokine secretion rate. Cell fate decision is then determined by rolling a dice and compared with the probability threshold of cell behavior (**S14 Fig**).

### Intracellular level

In this study, the proliferation rate of PCs was determined by two pathways. Our *hypothesis* is that: the androgen concentration in blood and gland will be sharply decreased after castration, so that prostate tumor cell proliferation will be supported by WNT5A or EGF-mediated androgen-independent pathway until the occurrence of CRPC (AR-reactivation). The ODE system for cell proliferation of PCs has been described in the above section. Except the ODE system was applied to model the intracellular signaling network in PC cells, Hill functions were used to simulate the signal transduction of other cells to calculate apoptosis and proliferation rates, and further determine the cell behaviors.

### Intercellular level

In response to the changes of WNT5A, EGF or DHT in its local mE, each prostate cancer cell will proliferate, migrate, become quiescent, or undergo death process. PCs secrete CSF1 to promote macrophage infiltration. Macrophage-derived EGF enhances tumor cell invasion [38]. TAMs also suppress the immune response of T cells by releasing the immunosuppressive factor, such as IL10 [88]. Similarly, the WNT5A-TRAIL positive loop between PCs and Tregs and the associated molecules are also considered as important modulating components for CRPC development and immune suppression. In addition, prostate tumor cells can be killed by CD8^+^ T cells. Treg cells can migrate towards CD8^+^ T cells locally and suppress the proliferation of these cells in a manner of cell cycle arrest or apoptosis [89].

#### Migration

A non-M-phase cell will migrate if it can find a free space nearby (**S15 Fig**). The ability of a cancer cell to undergo migration and invasion allows it to change position within the tissue. CD8^+^ T cells tend to move towards the places where tumor cells reside and try to eliminate residual cancer cells. Tumor cells can evade immune elimination through the loss of antigenicity and/or loss of immunogenicity and by coordinating an immunosuppressive microenvironment. Treg cells migrate to CD8^+^ T cells and suppress the proliferation of these effector cells. The migration was governed by space availability, migration speed, and stochastic effects using Hill functions and dice-casting simulation.

#### Programmed cytotoxic T cell response

A naïve T cell remains quiescent until it receives antigenic stimulation from dendritic cells through MHC-TCR interactions [55, 90]. After stimulation, naïve cells appear to be committed to a programmed response that causes them to divide and acquire effector functions [91]. For the first 19-24h, they do not replicate, but after this initial phase, they can rapidly undergo divisions with 7–10 generations [92]. After divisions, they acquire effector functions, such as cytotoxicity. In the ABM model, DC maturation started 12 hours later when they migrated into the lymph node. The initial immune response would be started 12 hours later after DC maturation. When T cells have received antigenic stimulation for 20 hours, they would rapidly divide (clonal expansion). For the clearance of cancer cells, each T cell might infiltrate to tumor space through the lymphatic vessel. After the stage of initial expansion, T cells continuously proliferate and to persist the population (T cell memory) [93]. We also simulated the activation and clonal expansion of naïve Tregs as CTLs in our model (**S1 Text**).

### Tissue level

#### Modeling of tumor angiogenesis

Tumor-induced angiogenesis is the process by which new blood vessels develop from existing vasculature, through endothelial cell sprouting and proliferation (**S16 Fig**). This process is essential for tumor growth and spread [94]. Solid tumors are known to progress through two distinct phases of growth: the avascular phase and the vascular phase [95]. The transition from the avascular stage to the vascular stage, wherein the tumor possesses the ability to invade surrounding tissue and metastasis to distant parts of the body, depends on the ability of the tumor to induce new blood vessels from the surrounding tissue to sprout towards and then gradually surround and penetrate the tumor, thus providing it with an adequate blood supply and microcirculation. Tumor-induced angiogenesis is believed to start when a small avascular tumor exceeds a critical diameter (~2mm) [95]. At this stage, the tumor cells lacking nutrients and oxygen. In response, the tumor cells secrete a number of tumor angiogenic factors (TAF), such as VEGF [25]. Endothelial cells (EC) respond to the TAF concentration, forming sprouts, proliferating and migrating towards the tumor [62]. It takes approximately 10–21 days for the growing network to link the tumor to the parent vessel [96, 97].

In our ABM model, we also simulated tumor-induced angiogenesis. As shown in **S4 Fig**, the prostate tumor at the start point is in the avascular phase, and the initial tumor size is around ~1mm radius. With the distribution of VEGF in the tumor microenvironment [25], sprout branching and anastomosis were developed from an existing parent vessel (**S16 Fig**). The generation of new sprout (branching) occurs only from existing sprout tips. The newly formed sprouts are unlikely to branch immediately and that there must be a sufficient number of endothelial cells, near the sprout tip, for new sprouts to form (branching age is 18 hours [62]). We assume that the density of endothelial cells required for branching is inversely proportional to the concentration of TAF. In order to accomplish vascularization, the endothelial cells must proliferate and subsequently migrate the whole distance to the tumor [98]. After 4 weeks, the simulated prostate tumor will reach to ~4mm radius [99], and it will undergo castration. The details related to the modeling of tumor-induced angiogenesis were described in **S1 Text**.

#### T-cell activation and infiltration

Several studies have reported that tumor-associated antigens can induce T cell responses [100] and the production of antibodies, indicating that with the optimum balance of immune effectiveness over immune suppression [58, 59], and the immune system can fight cancer.

In this study, the proposed model simply simulated the cycle of generating an antitumor response. Dendritic cells engulf antigens from dying prostate tumor cells. As these dendritic cells migrate to the draining lymph node, they present the prostate-specific antigen (PSA) to naïve T cells (CD8^+^ effector T cells, regulatory T cells, etc.) for their clonal expansion and differentiation in the lymph node [101, 102]. Activated CD8^+^ effector T cells migrate to the tumor bed through the blood vessels or the lymphatic vessels [55]. When CD8^+^ T cells encounter tumor cells, they initiate programmed events leading to tumor cell death. Tregs similarly return to the tumor and suppress effector T cell killing efficiency to guard against overt inflammation and normal tissue damage. Within 2 days, these DCs lose their motility as they become integrated into the network of lymph-node DCs and die rapidly [56].

**Fig 3** defines the entire simulated microenvironment consisting of tumor space and lymph node, which are connected with one lymphatic vessel. There is also a lymphatic vessel generated from lymph node, and it involves in the cycle of peripheral blood. In our model, activated CTLs and Tregs are migrated mainly through lymphatic vessel, moved to and accumulated in tumors. Each T cell agent is randomly selected and then infiltrated into the tumor tissue; its coordinates will be updated immediately. A schematic illustration of tumor growth and immune infiltration simulated by our HMSM model is displayed in **S17 Fig**.

### Model implementation

The framework of the ABM model was designed using the conception of “Object-Oriented Programming” and achieved with C++. The ODE system of intracellular signaling pathways in PC was established with C and solved by the Fortran ODE Solver (DLSODE [103]), and called in the ABM model of HMSM (**S1.10 Text**). The ABM model was debugged and implemented under Linux environment on the cluster platform of Demon in Wake Forest Baptist Medical Center and Texas Advance Computing Center (TACC). All of the parameters in the ABM model were tuned by running the system 100 times for each candidate solution. The model with optimal parameters should fit the training data well. For addressing the stochastic results from the ABM, we evaluated the model outcomes after replicating simulations (repeat 100 times) on a fixed model. Both average and standard deviation were used to present the results.

## Supporting information

S1 Text Development of HMSM model. (DOCX)

S1 Fig The calculation procedures for identifying overexpressed ligand-receptor gene pairs. (TIF)

S2 Fig The inferred cell-cell interactions between PCs and Tregs from public GEO datasets. Twenty-three overexpressed ligand genes and 39 overexpressed receptor genes were identified from the dataset GSE46218, respectively. Eighteen overexpressed ligand genes and 26 receptor genes were identified from the dataset GSE38043. Based on the public ligand-receptor interaction database (iRefWeb) with a high confidence socore, a potential interaction pair was found: Treg→WNT5A→PC, and PC→TRAIL→Treg.
(TIF)

S3 Fig RNA-seq analysis showing the effect of WNT5A on the expression of a group of genes in 22RV1 cells. 22RV1 cells were treated with 250ng WNT5A and RNA samples collected at 1, 3, 7, and 24 hours.
(TIF)

S4 Fig The key biological events included in the HMSM model. (TIF)

S5 Fig A simulation example of CSF1 profiling before/after castration. Two slices are presented: Y = 40, and Y = 50. Y is the Y axis (*0≤Y≤100*).
(TIF)

S6 Fig A simulation example of EGF profiling before/after castration. Two slices are presented: Y = 40, and Y = 50. Y is the Y axis (*0≤Y≤100*).
(TIF)

S7 Fig A simulation example of VEGF profiles before/after castration. Two slices are presented: Y = 40, and Y = 50. Y is the Y axis (*0≤*Y*≤100*).
(TIF)

S8 Fig The flow chart of our study. (TIF)

S9 Fig Heterogeneous expressions of AR-related proteins under three conditions in dataset GSE67980. Primary tumor represents that the human tumor tissues were from PCa patients without treatment. CRPC (enzalutamide-naïve) indicates the human tumor tissues were from the PCa patients with CRPC occurrence who had not received enzalutamide. CRPC (progressed on enzalutamide represents the human tumor tissues were from the PCa patients who had received enzalutamide treatment after CRPC occurrence.
(TIF)

S10 Fig Parameter sensitivity analysis. Sensitivity analysis was performed by measuring the impact of a small perturbation (5% increase) of individual 34 key parameters on the tumor cell (PC) population.
(TIFF)

S11 Fig Testing the impact of initial cell numbers on model variability. The fold change of tumor growth on day 35 after castration was examined by a 5% or 10% increase in the initial PC or TAM population. “Control” denotes the simulation without perturbation. The results are comparable with those in **Fig 6L**. The variability of the average value is in the range of -1.1139% to 1.7024%.
(TIF)

S12 Fig Testing the impact of cut-off values in the ABM rules on model variability. The fold change of tumor growth on day 35 (5 weeks) after castration was examined by a 5% increase or decrease in the individual cut-off value in the migration rules. “Control” denotes the simulation without perturbation. The results are comparable with those in **Fig 6L**. The variability of the average value for increase or decrease is in the range [-1.07%, 0.12%] and [-1.94%, 1.26%], respectively.
(TIF)

S13 Fig Time course simulation. (A) The fold change of TAM population after castration relative to pre-castration. (B) The fold change of CSF1 expression after castration relative to pre-castration. (C-D) the fold change of IL10 and VEGF expressions after castration relative to pre-castration. (E-F) The fold change of TAM population and VEGF expression after the treatment with castration plus PLX comparted to castraton only. (G) The fold change of Treg population in lymph nodes after castration relative to pre-castration. (H) The fold change of Treg population in lymph nodes after the treatment with castration plus IL-2 neutralization compared to castration only. (I-J) The fold change of CD8+ and Treg population in tumor space after castration relative to pre-castration. (K) The fold change of tumor growth after castration relative to pre-castration. (L) The fold change of tumor growth after treatment with castration plus PLX compared to pre-castration. (M) The fold change of tumor growth after treatment with castration plus EGFR inhibitor to castration only.
(TIF)

S14 Fig The stochastic simulation of cell behaviors. (TIF)

S15 Fig A sketch showing how spatial dispersal is implemented. A cell at position *X*_*i*_ searches for the candidate locations within the distance *R*, and several empty positions (red dots) are identified. The probability (*M*) of a cell moving from *X*_*i*_ to *X*_*j*_ was determined by: 1) the moving offset (|*d*_*i*_|); 2) the number of occupied cells (blue dots) around the new position; and 3) the type of the occupied cells. In our model, *R* equals 2 for migration, and 1 for proliferation.
(TIF)

S16 Fig The strategy for generating sprouts during model initialization. If 0≤*x*≤14 or 86≤*x*≤100, *ρ* = 0; otherwise, *ρ* follows a normal distribution (14<*x*<86).
(TIFF)

S17 Fig A schematic illustration of tumor growth and immune infiltration simulated by our HMSM model. (TIF)

S1 Table Overexpressed ligands and receptors in PCs inferred from GSE46218 (P-value<0.05). (DOCX)

S2 Table Overexpressed ligands and receptors in Tregs inferred from GSE38043 (P-value<0.05). (DOCX)

S3 Table Enriched pathways associated with significantly expressed genes after WNT5A treatment (Top 10 enriched pathways obtained from KEGG, P-value<0.05). (DOCX)

S4 Table Enriched genes of TAM and PCa cells identified from our RNA-seq data (FC>1.3). (DOCX)

S5 Table The parameters in ODE system optimized by GA algorithm. (DOCX)

S6 Table The inferred parameters for agent-based model in HMSM. (DOCX)

S7 Table The sequences of the paired sense and antisense primers for human Antigen receptor, TNF-10, FTZ1 and β-actin. (DOCX)

S1 Data The top-ranked enriched ligand or receptor-associated genes in LnCAP, 22RV1, and TAM cells. (XLSX)

S2 Data The experimental observation and *in silico* prediction of HMSM model. (DOCX)
