# Supplementary material for: Systematically understanding the immunity leading to CRPC progression
Source: PLoS Comput Biol. 2019 Sep 10;15(9):e1007344. doi: 10.1371/journal.pcbi.1007344 (PMC6754164; doi:10.1371/journal.pcbi.1007344)
Supplement: S1 Text — (DOCX) [file pcbi.1007344.s001.docx]

**S1 Text. Development of HMSM model**

**1.1 Simulated microenvironment**

In this study, we defined five types of cell agents in the HMSM model to represent PC, TAM, CD8^+^ (CTL), Treg, and EC. We initialized the tumor microenvironment as a 200*100*100 3D matrix, which is consisting of two parts: tumor space (100*100*100 cube) and lymph node space (100*100*100 cube), where 1 grid equals to 0.2 mm. The structure of the simulated system is shown in **Fig 3**. At the beginning, the mixed population of PC and TAM were initialized in the center of the tumor space as a sphere with radius 5 grids (initial size has a diameter around 2mm (1)). And the mixed population of CTL and Treg were initialized in the center of the lymph node as an ellipsoid and three axes are 6, 8, and 10 grids. The numbers of each type of cell are 200 (PC), 100(TAM), 2 (CD8 in the lymph node), 2 (Treg in the lymph node). Initially, new sprouts (ECs) occur from the main blood vessel (**Fig 3**). We assumed that 5-10 branches will be randomly generated from the main blood vessels and will grow new vessels toward the tumor (2).

**1.2 The computational framework HMSM**

Our HMSM model is a hybrid model, which integrates the ODE system into an agent-based model (ABM). The basic framework of ABM used in HMSM was developed in our previous HABM model (3). The Markov Chain Monte Carlo approach was used to simulate cell behaviors of each individual cell. As shown in **S14 Fig**, cell behaviors were simulated by probability-based rule implementation. A cell sensed the growth factors or drug doses from its neighborhood, processed them with signaling pathways (via ODE systems or Hill functions), and outputted the changes of probabilities of cell behaviors including cell proliferation rate, apoptosis rate, migration rate, and cytokine secretion rate. Cell decision was then determined by rolling a dice and compared with the probability of given cell behavior. Details of cell behaviors for each type of cell agent as well as the corresponding rule was introduced in the following sections.

**1.3 Cell proliferation**

The cell decision-making process was defined by agent rules, and the stochastic feature of the decision of an individual cell was realized by dice rolling simulation. Here, we used the cell decision-making of entering cell cycle as an example to elaborate the algorithm. Cell proliferation rules were defined according to proliferation rates with a dice $C_{rand}\epsilon[0,1]$. A cell cycle in our simulated system is 26 hours. If the dice fell into the interval [0, $P_{prol}$], the cell entered cell cycle and started to proliferate; otherwise, stayed quiescent. Four cell cycle phases (G0/G1, S, G2, and M) were defined (3). Cells kept migrating during the first three phases, while tried to find a location to proliferate after entering the M phase. The duration of stage G0/G1 (10 hours) was determined by the Monte Carlo simulation (dice rolling) process, the S phase lasted for 6 hours (3 time steps), and the G2 phase would not exceed 6 hours (3 time step). Based on the above rules, the calculations of each type of cell agent were described as follows.

The proliferation rate of a prostate cancer cell at the location $(i,j,k)$ was described as shown in Eq. (1):

${Prol\_PC}_{ijk}=f*\frac{\mathrm{ACcrpc}}{ACcrp+H_{D}}*ADPCpro0+AIPCpro0*\left( \frac{1+P_{ODE}}{K_{v0}} \right)^{n}$ (1)

Where ADPCpro0 and AIPCpro0 are the initial proliferation rates of PCs via androgen-dependent and independent pathways, respectively. Logical variable $f$ is a flag to control the effects induced by androgen dependent pathways. After castration, the value of $f$ is equal to 0 before AR is reactivated; otherwise, 1. ACcrpc represent the fold change of androgen concentration, which was decreased after castration (default: $H_{D}=1$). The function $P_{ODE}$ was defined as $P_{ODE}$(${WNT5A}_{ijk}$, $D1, {EGF}_{ijk}, D2$), which indicates the effects of WNT5A-EGF/AR signaling pathway on proliferation. The local concentration of WNT5A and EGF at the location $(i,j,k)$ are denoted as ${WNT5A}_{ijk}$ and ${EGF}_{ijk}$, respectively. D1 and D2 represent the inhibitors of WNT5A and EGF pathways, respectively. $P_{ODE}$ can be calculated by our developed ODE system (see the details in **Materials and Methods**).

The proliferation rate of a TAM at the location $(i,j,k)$ was defined in Eq. (2):

${Prol\_M}_{ijk}=TAMpro0+TAMCSFpro0*\left( 1-PLX \right)*[\frac{{CSF}_{ijk}/K_{v4}}{{{1+CSF}_{ijk}/K}_{v4}}]$ (2)

In Eq. (2), TAMpro0 is the initial proliferation rate of TAM cells, and TAMCSFpro0 is the proliferation rate induced by activated CSF1R. The logical flag PLX (0 or 1) control the presence of CSF1R inhibitor (PLX3397).

Treg residents in tumor space can be regulated by IL10 and TRAIL. Eq. (3) defines the proliferation rate of Tregs in the tumor:

${Prol\_T}_{ijk}=Tregpro0*\left( 1+\frac{\left( \frac{{IL10}_{ijk}}{K_{v6}} \right)n}{1+\left( \frac{{IL10}_{ijk}}{K_{v6}} \right)n} \right)*\left( 1+\frac{\left( \frac{{TRAIL}_{ijk}}{K_{v7}} \right)n}{1+\left( \frac{{TRAIL}_{ijk}}{K_{v7}} \right)n} \right)$ (3)

Where Tregpro0 is the initial proliferation rate of Treg cells. Previous studies reported that IL-2 is mainly generated from CD8 cells, and is required for functional maturation of Treg (4-6). Therefore, the proliferation rate of Tregs in lymph node was defined as:

${Prol\_T}_{ijk}=Tregpro0$*$\left( 1+[\left( 1-PT \right)*\frac{\left( \frac{{IL2}_{ijk}}{K_{v5}} \right)n}{1+\left( \frac{{IL2}_{ijk}}{K_{v5}} \right)n}] \right)$ (4)

Where *PT* is the IL-2 antibody. The proliferation rate of CD8^+^ T cell (CTL) in tumor space was not only suppressed by the population of Tregs (7) but also was enhanced by IL-10 (8). Eq. (5) represents the proliferation rate of a CTL in the location $(i,j,k)$:

${Prol\_C}_{ijk}=CTLpro0*\left[ \frac{\left( \frac{R}{Kr} \right)n}{1+\left( \frac{R}{Kr} \right)n} \right]+CTLRpro0*\left[ \frac{1}{1+\left( \frac{{IL10}_{ijk}}{K_{v6}} \right)n} \right]$ (5)

Where CTLpro0 and CTLRpro0 are the initial proliferation rate and the proliferation rate induced by IL-10, respectively. The suppressive effect of Tregs on CD8^+^ T cells was described with variable $R$, which determined by the population ratio of Treg against CD8^+^. Our previous study inferred the formulation of function $R$ (3), which is shown as in Eq. (6-7):

$R=0.82-3.03*\frac{r-0.0659}{3.5609+(r-0.0659)}$ (6)

$r=\frac{Treg population}{CTL population}$ (7)

According to the above formula (6-7), we suggest that Treg expansion will induce CTL-mediated immune response by inhibiting CTL proliferation.

The proliferation of ECs during angiogenesis was defined as the migration of sprout tips toward tumor (9). The related details were described in the following section.

**1.4 Cell apoptosis**

At each time step, if the apoptosis rate of a cell agent was lesser than the threshold, it would start apoptosis. Each cell takes 10 time steps to finish apoptosis and was then absorbed. The cell apoptosis is not only regulated by cell-death-related signaling pathways but also is affected by the cytokines in the tumor microenvironment.

The apoptosis rate of PC was defined in Eq. (8):

${APOP\_PC}_{ijk}=ADPCapop0*\left( 1+\frac{\left( \frac{R}{Kr} \right)n}{1+\left( \frac{R}{Kr} \right)n} \right)+AIPCapop0*\left( \frac{1}{1+{(\frac{Pode}{K_{v1}})}^{n}} \right)$ (8)

where ADPCapop0 denotes the initial apoptosis rate of PC before castration and AIPCapop0 represents the apoptosis rates of PC after castration. According to Eq. (8), we can clearly see that the apoptosis of PCs can be increased by CTL-mediated lysis, but might be reduced by WNT5A and EGF expression. In the meantime, Tregs have been shown to inhibit tumor-specific T cell functions, such as the cytotoxic effects of CTL, by suppressing the proliferation or inducing the apoptosis of CTL (7). The apoptosis rate of CTL was defined as Eq. (9):

${APOP\_C}_{\mathrm{ijk}}=CTLapop0+CTLRapop0*\left( \frac{\left( \frac{IL10}{K_{v6}} \right)n}{1+\left( \frac{IL10}{K_{v6}} \right)n} \right)*(\frac{1}{1+\left( \frac{R}{Kr} \right)n})$ (9)

Where CTLapop0 is the initial apoptosis rate and CTLRapop0 is the apoptosis rate induced by Treg expansion. In addition, the apoptosis rates of Tregs and TAM were defined in Eq. (10) and Eq. (11), respectively. *PT* denotes anti-IL-2, which affects the Treg expansion in the lymph node. The details of the parameters used in Eq. (10-11) were described in **S6 Table**.

${APOP\_T}_{ijk}=Tregapop0*\left( \frac{1}{1+\left( \frac{IL10}{K_{v6}} \right)n} \right)*\left( \frac{1}{1+\left( \frac{TRAIL}{K_{v7}} \right)n} \right)+TregSigapop0*PT$ (10)

${APOP\_M}_{ijk}=TAMapop0+TAMCSFapop0*PLX$ (11)

Considering that most of growth factors in our model are distributed within tumor space, we assume that the apoptosis rates of CTLs and Tregs are initial apoptosis rates (see **S6 Table**).

**1.5 T cell infiltration**

As the results of the initial immune response, naïve T cells (CD8 and Treg) can rapidly undergo a number of divisions once every 5-8 h (10, 11). After activation, a small subpopulation continues to proliferate and persists as memory cells (12). In our model, the average cycle time for a T cell to division is 6h and the maximum generation will up to 4. To response the tumor growth, a number of activated immune cells will infiltrate from the lymph node to tumor space through lymph vessel (13). As shown in **S17 Fig**, a T cell in the lymph node can be randomly selected with a probability and directly move to the tumor side for an anti-tumor immune response if there is free space around the left side of the lymph vessel. After a CTL arrived at the tumor area, which involved in the lysis or apoptosis of tumor cells. In the meantime, Treg infiltration will induce the suppression of CTL proliferation.

**1.6 Angiogenesis model**

The model of EC migration given below describes how capillary sprouts emerging from a parent vessel migrate towards a tumor, leading to the formation of a vascular network that supplies nutrients for continued tumor development. The proliferation of the endothelial cells at the capillary tips and branching at capillary tips are implemented in the model. The probability of branching increases as the TAF concentration increases (14). **Fig 3** gives a schematic illustration of branching, which we will model in our system.

We assumed that the generation of new branching occurs only from existing sprout tips. The newly formed sprouts are unlikely to branch immediately and there must be a sufficient number of ECs, near the sprout tips, for new sprouts to form (15). The generation of new sprouts positively depends on TAF concentration and also associated with the distance to the tumor. For each sprout tip cell, we check whether the following conditions are satisfied for generating a new sprout:

1. The age of the current sprout is greater than a threshold branching age $nf$. New sprouts aren’t able to branch until they mature for a length of time at least equal to $nf$. In this study, $nf$ is defined as 18 hours.
2. There are at least two free sites locally for two new sprouts to form at a sprout tip.
3. The EC density is greater than a threshold level $nd$. Initially, very little branching occurs near the parent vessel, but as the ECs migrate closer to the tumor the number of new sprouts slowly increases.

$nd=\left\{ \begin{aligned} 0.1, if \left( Lx,Ly,Lz<0.2*\frac{G}{2} \right)||\left( Lx,Ly,Lz>G-0.2*\frac{G}{2} \right) \\ 0.2, if \left( Lx,Ly,Lz<0.4*\frac{G}{2} \right)||\left( Lx,Ly,Lz>G-0.4*\frac{G}{2} \right) \\ 0.3, if \left( Lx,Ly,Lz<0.6*\frac{G}{2} \right)||\left( Lx,Ly,Lz>G-0.6*\frac{G}{2} \right) \\ 0.4, if \left( Lx,Ly,Lz<0.7*\frac{G}{2} \right)||\left( Lx,Ly,Lz>G-0.7*\frac{G}{2} \right) \\ 1, else \end{aligned} \right.$ (12)

In Eq. (12), (*Lx, Ly, Lz*) represents the current sprout tip, and *G* is the length of the cube (*G*=100 grids).

If the above conditions are satisfied, a random number $r_{1}$ is generated, if $r_{1}<probrch$, then two new endothelial cells will be generated on the direct neighbors of the current tip and form a branching; otherwise, the sprout tips will continually migrate toward the tumor (see the next section). The final probability of branching $probrch$ was defined as shown in Eq. (13).

$probrch=(0.8+0.2*\left( cur_{V}/{max}_{V} \right))*nd$ (13)

where $cur_{V}$ and ${max}_{V}$ denote the local and maximal concentration of VEGF.

Before model initialization, there is a pre-existing vasculature (see the left panel in **Fig 3**). At the beginning of initialization, the ABM model randomly generated tips on the vasculatures. The new small vessels sprout from those tips and grow towards the center of the tumor. The probability ($\rho$) for generating the sprout from a tip was defined as a distribution shown in **S16** **Fig**. Obviously, the location x ($0\leq x\leq100$) closing to the center of the distribution has larger possibility to grow a new vessel.

**1.7 Cell migration**

A non-M-phase cell at position *P* will migrate if it can find free space in its neighborhood (**S15 Fig**). We assumed that PCs and TAMs migrate toward each other and promote the effects on PC growth. For the CD8^+^ T cells (CTLs), they tried to migrate toward prostate tumor cells and to induce the lysis of target cells. As to Tregs, they prefer to migrate to the CTL population and to induce the cell cycle arrest or apoptosis of those cells. As reported in our previous studies, all the unoccupied positions ($P_{ijk}$) within a radius $r_{max}$ from the original position $P_{0}$ were scored and ranked (3):

$r_{max}=\left\lfloor\frac{2(1+4D*\Delta t)}{10} \right\rfloor+1$ (14)

Where $D$ was the basic migration speed index, $\Delta t$ is the time step (2 hours). In addition, $p\left( r_{l} \right)$ was defined as the visiting chance of position $P_{ijk}$ with distance $r_{l}$ ($r_{l}\leq r_{max}$):

$p\left( r_{l} \right)=\frac{1}{4\pi D\Delta t}exp(\frac{-{r_{l}}^{2}}{4\pi D\Delta t})$ (15)

Now, we firstly introduce the rules for PC migration. All the candidate unoccupied locations were ranked with Eq. (16):

$R_{l}=p\left( r_{l} \right)*C_{EC}*C_{PT}*C_{CTL}*V_{l}$ (16)

In Eq. (16), variable $C_{EC}$ denotes if the candidate location $P_{ijk}$ exists an immediate neighbor as an endothelial cell. Nearby blood vessels receive the signals from the tumor mass and begin to grow new vessels toward the source. $C_{PT}$ indicates if PCs prefer to move toward PCs or TAMs. Also, the variable $C_{CTL}$ represents if there is an immediate neighbor of $P_{ijk}$ as a CD8^+^ T cell. The values of $C_{EC}$, $C_{PT}$, and $C_{CTL}$ were defined by Eq. (17-19).

$C_{EC}=\left\{ \begin{aligned} 1.0, at least a neighbor is EC \\ 0.25, \mathrm{otherwise} \end{aligned} \right.$ (17)

$C_{PT}=\left\{ \begin{aligned} 1.0, at least a neighbor is PC or TAM \\ 0.50, \mathrm{otherwise} \end{aligned} \right.$ (18)

$C_{CTL}=\left\{ \begin{aligned} 0.5, at least a neighbor is CTL \\ 1, \mathrm{otherwise} \end{aligned} \right.$ (19)

In addition, $V_{l}$ described prostate cells try to avoid loneliness as well as crowdedness (see Eq. (20)).

$V_{l}=\left\{ \begin{aligned} 1/8, P_{ijk} has 5-6 neighbor cells \\ 1/4, P_{ijk} has 3-4 neighbor cells \\ 1, P_{ijk} has 1-2 neighbor cells \\ 1/16, P_{ijk} has 0 neighbor cells \end{aligned} \right.$ (20)

After calculated the scores ($R_{l}$) of all the candidates, and then ranked them and selected the final location to migrate by dice casting (see the Eq. 17-18 in (3)).

For TAM agent, the migration rules were defined with the above ways. The candidate locations for a TAM migrate were calculated with Eq. 21.

$R_{l}=p\left( r_{l} \right)*C_{PT}{*V}_{l}$ (21)

Where $C_{PT}$ denotes if the current candidate location exists an immediate neighbor as PC or TAM. The definition of $p\left( r_{l} \right)$, $C_{PT}$ and $V_{l}$ in Eq. (21) were described above.

For a CD8^+^ T cell, it will migrate towards PCs for inducing the apoptosis of target cells rather than empty position. The candidate rank was calculated with Eq. (22).

$R_{l}=p\left( r_{l} \right)*C_{PC}{*C_{Treg}*V}_{l}$ (22)

In Eq. (22), variable $C_{PC}$ denotes if the current candidate location exists an immediate neighbor as a prostate cancer cell. And $C_{Treg}$ represents if there is an immediate neighbor as a Treg cell. The definition of $C_{PC}$ and $C_{Treg}$ were shown in Eq. (23) and (24), respectively.

$C_{PC}=\left\{ \begin{aligned} 1.0, at least a neighbor is PC \\ 0.25, \mathrm{otherwise} \end{aligned} \right.$ (23)

$C_{Treg}=\left\{ \begin{aligned} 0.5, at least a neighbor is Treg \\ 1.0, \mathrm{otherwise} \end{aligned} \right.$ (24)

As to the regulatory T cell (Treg), it will migrate towards CD8^+^ T cell and try to suppress the proliferation of the effector T cells. The candidate rank was calculated with Eq. (25).

$R_{l}=p\left( r_{l} \right)*C_{PT}{*C_{CTL2}*V}_{l}$ (25)

In Eq. (25), variable $C_{CTL2}$ denotes if the current candidate location exists an immediate neighbor as CTL cell (see Eq.(26)).

$C_{CTL2}=\left\{ \begin{aligned} 1, at least a neighbor is CTL \\ 0.5, \mathrm{otherwise} \end{aligned} \right.$ (26)

Finally, the migration of ECs is defined as the proliferation of sprout tips before branching (9, 16). If the branching conditions were not satisfied, we move a tip endothelial cell toward the tumor by rolling a dice (15). Endothelial cell doubling time is about 18 hours, and we modeled the process of cell division by assuming that a sprout tip divides into two cells every 18 hours. Therefore, our model searches a free location for the current sprout tip, and then implements the division (two daughters will locate at the original site and new site, respectively). The candidate-unoccupied locations were ranked as Eq. (27):

$R_{l}=p\left( r_{l} \right){*V_{A}*V}_{l}$ (27)

In Eq. (27), $V_{A}$ denotes the average concentration of the immediate neighbors of current candidate location, which indicates that blood vessels receive the nearby signals of growth factors (e.g. VEGF) and begin to grow new vessels toward the source.

**1.8 Dynamic expression of growth factors**

The dynamic expression profile (concentration) of growth factors (WNT5A, TRAIL, CSF1, EGF, VEGF, IL2, IL10, etc.) in the 3D simulated tumor microenvironment was defined as Eq. (28).

$S_{ijk}\left( t+1 \right)=\left( 1-DEG \right)*\left[ {(S}_{ijk}\left( t \right)+\Delta S(t+1))*\left( 1-\omega\right)+\frac{\omega}{6}\sum_{l=1}^{6} S_{ijk}^{l}(t+1) \right]$ (28)

where $S_{ijk}\left( t+1 \right)$ is the concentration of the factor on the position ($i,j,k$) updated at the time step $t+1$, and $\omega$ is the smooth constant. $\Delta S(t+1)$ denotes the growth factor secretion from cells on the position ($i,j,k$) at time step $t+1$. The value $S_{ijk}^{l}(t+1)$ indicates the concentration of a location, which is one of six immediate neighbors of position ($i,j,k$). The constant $DEG$ represents the degrading rate of growth factors.

**1.9 Parameter tuning of ABM model**

Our parameter tuning includes the following three steps.

**First step**: Initialization of parameters for ABM model.

**Second step**: Repeated the simulation of ABM model for 100 times. After replicating simulations, we calculated the average value and standard deviation for the cell population, cytokine expression, etc. And then we adopted the average relative error (ARE) between experimental and predicted values to judge the goodness of fit. The calculation of ARE is shown as following:

$ARE=\frac{1}{K}\sum_{i=1}^{K} \frac{\left| P_{i}-E_{i} \right|}{E_{i}}$ (29)

where K is the total number of all fitted data points. $E_{i}$ and $P_{i}$ are the $i$-th experimental and predicted data, respectively. For simplicity, we used the mean of each variable ($E_{i}$ and $P_{i}$) to estimate ARE (see **S2 Data**). In our study, all the $E_{i}$ and $P_{i}$ were fold change values (**S2 Data, Fig 6**), therefore, we set 0.15 as the threshold of the acceptable errors (ARE$\leq$0.15).

**Third step**: if the error is acceptable, the tuning process can be stopped; otherwise, we manually tuned the parameters based on the values tested in the second step, and then repeated the second step. If we obtained multiple acceptable solutions, we selected the cases with the lowest error. It is most desirable to test each model with additional new validation data and then choose the optimal parameter set.

Based on previous studies (3, 17), 100-200 simulations in the second step is an acceptable number for obtaining steady results of ABM model. Therefore, the aim of parameter tuning is to find a suitable parameter set.

**1.10 Setting-up the HMSM model in the Linux system**

Firstly, we used Matlab to set the ODE functions of signaling pathways. Secondly, we collected the proteomics data from our in vitro experiments. Thirdly, we estimated the parameters involved in ODEs with GA algorithm in Matlab (**S5 Table**). Once the parameters were fixed, the outcome of the ODE system can be determined for a given input. The results shown in **Fig 5E-I** were simulated in Matlab and done outside of the ABM model.

And then, we re-build the ODE system with C codes by using the ODE formulas and the optimal parameters estimated using the GA algorithm mentioned above. Therefore, the C-based ODEs is capable of reproducing the same results as the Matlab version. The C-based ODE system was solved by the Fortran ODE Solver (3) (compiled with gfortran). This C file of the ODE system and all C++ files of ABM model are finally compiled with gcc and g++, respectively. Finally, all the .o files were combined by using gfortran to generate one executable file.

**Supplementary Reference**

1. Macklin P, McDougall S, Anderson ARA, Chaplain MAJ, Cristini V, Lowengrub J. Multiscale modelling and nonlinear simulation of vascular tumour growth. Journal of Mathematical Biology. 2009;58(4-5):765-98.

2. Carmeliet P, Jain RK. Angiogenesis in cancer and other diseases. Nature. 2000;407(6801):249-57.

3. Ji Z, Su J, Wu D, Peng H, Zhao W, Nlong Zhao B, et al. Predicting the impact of combined therapies on myeloma cell growth using a hybrid multi-scale agent-based model. Oncotarget. 2017;8(5):7647-65.

4. Tang S, Moore ML, Grayson JM, Dubey P. Increased CD8(+) T-cell Function following Castration and Immunization Is Countered by Parallel Expansion of Regulatory T Cells. Cancer Research. 2012;72(8):1975-85.

5. Owen DL, Mahmud SA, Vang KB, Kelly RM, Blazar BR, Smith KA, et al. Identification of Cellular Sources of IL-2 Needed for Regulatory T Cell Development and Homeostasis. J Immunol. 2018;200(12):3926-33.

6. de la Rosa M, Rutz S, Dorninger H, Scheffold A. Interleukin-2 is essential for CD4+CD25+ regulatory T cell function. Eur J Immunol. 2004;34(9):2480-8.

7. Bauer CA, Kim EY, Marangoni F, Carrizosa E, Claudio NM, Mempel TR. Dynamic Treg interactions with intratumoral APCs promote local CTL dysfunction. J Clin Invest. 2014;124(6):2425-40.

8. Wang L, Liu JQ, Talebian F, Liu Z, Yu L, Bai XF. IL-10 enhances CTL-mediated tumor rejection by inhibiting highly suppressive CD4(+) T cells and promoting CTL persistence in a murine model of plasmacytoma. Oncoimmunology. 2015;4(7):e1014232.

9. Sholley MM, Ferguson GP, Seibel HR, Montour JL, Wilson JD. Mechanisms of neovascularization. Vascular sprouting can occur without proliferation of endothelial cells. Lab Invest. 1984;51(6):624-34.

10. Kaech SM, Ahmed R. Memory CD8+ T cell differentiation: initial antigen encounter triggers a developmental program in naive cells. Nat Immunol. 2001;2(5):415-22.

11. van Stipdonk MJ, Lemmens EE, Schoenberger SP. Naive CTLs require a single brief period of antigenic stimulation for clonal expansion and differentiation. Nat Immunol. 2001;2(5):423-9.

12. Murali-Krishna K, Lau LL, Sambhara S, Lemonnier F, Altman J, Ahmed R. Persistence of memory CD8 T cells in MHC class I-deficient mice. Science. 1999;286(5443):1377-81.

13. Adams JL, Smothers J, Srinivasan R, Hoos A. Big opportunities for small molecules in immuno-oncology. Nat Rev Drug Discov. 2015;14(9):603-22.

14. Muthukkaruppan VR, Kubai L, Auerbach R. Tumor-induced neovascularization in the mouse eye. J Natl Cancer Inst. 1982;69(3):699-708.

15. Sun XQ, Zhang L, Tan H, Bao JG, Strouthos C, Zhou XB. Multi-scale agent-based brain cancer modeling and prediction of TKI treatment response: Incorporating EGFR signaling pathway and angiogenesis. Bmc Bioinformatics. 2012;13.

16. Paweletz N, Knierim M. Tumor-related angiogenesis. Crit Rev Oncol Hematol. 1989;9(3):197-242.

17. Su J, Zhang L, Zhang W, Choi DS, Wen J, Jiang B, et al. Targeting the biophysical properties of the myeloma initiating cell niches: a pharmaceutical synergism analysis using multi-scale agent-based modeling. PLoS One. 2014;9(1):e85059.
