# Supplementary figures and images for: Systematically understanding the immunity leading to CRPC progression
Source: PLoS Comput Biol. 2019 Sep 10;15(9):e1007344. doi: 10.1371/journal.pcbi.1007344 (PMC6754164; doi:10.1371/journal.pcbi.1007344)

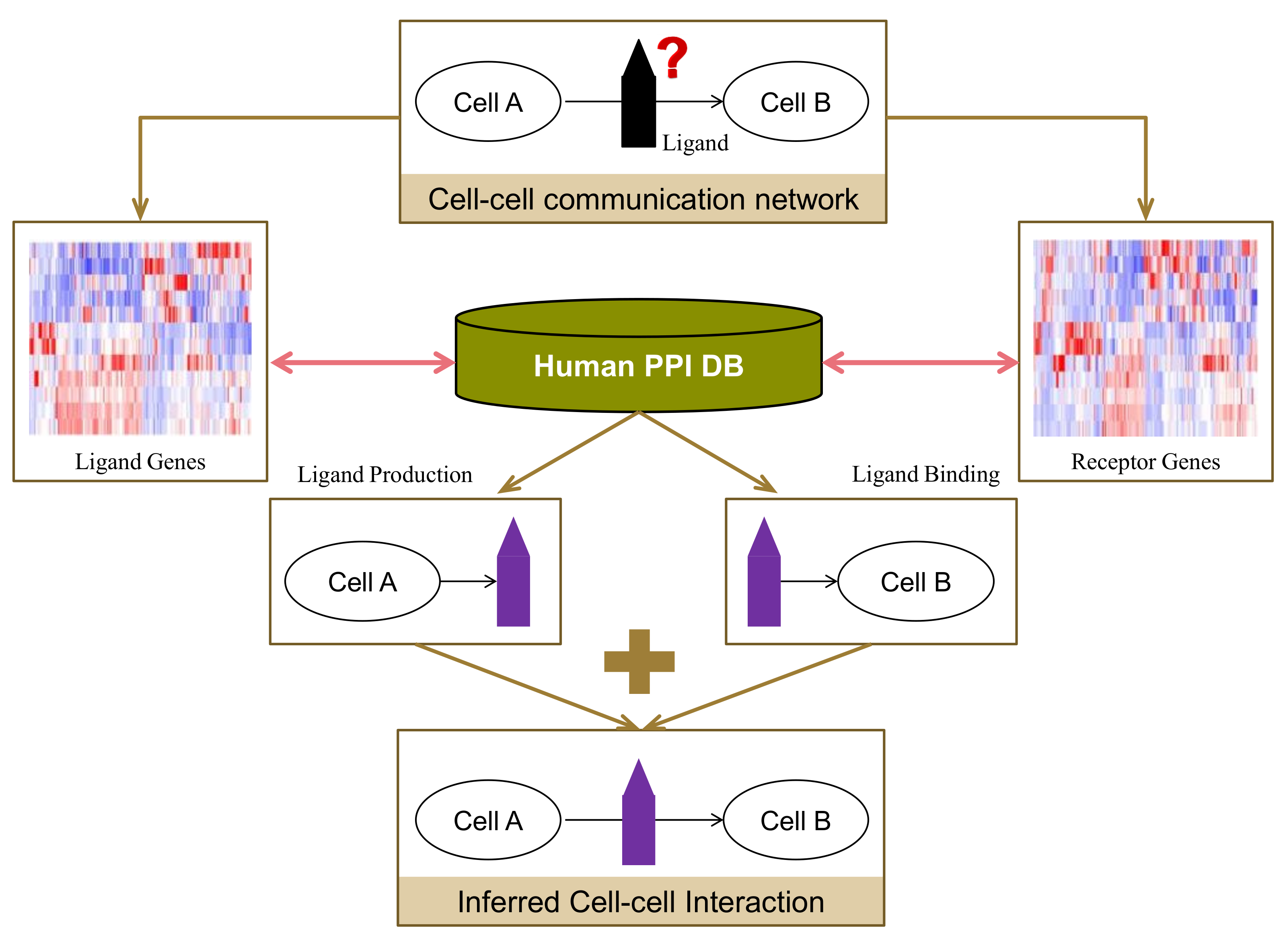

Supplement: S1 Fig — (TIF) [file pcbi.1007344.s002.TIF]

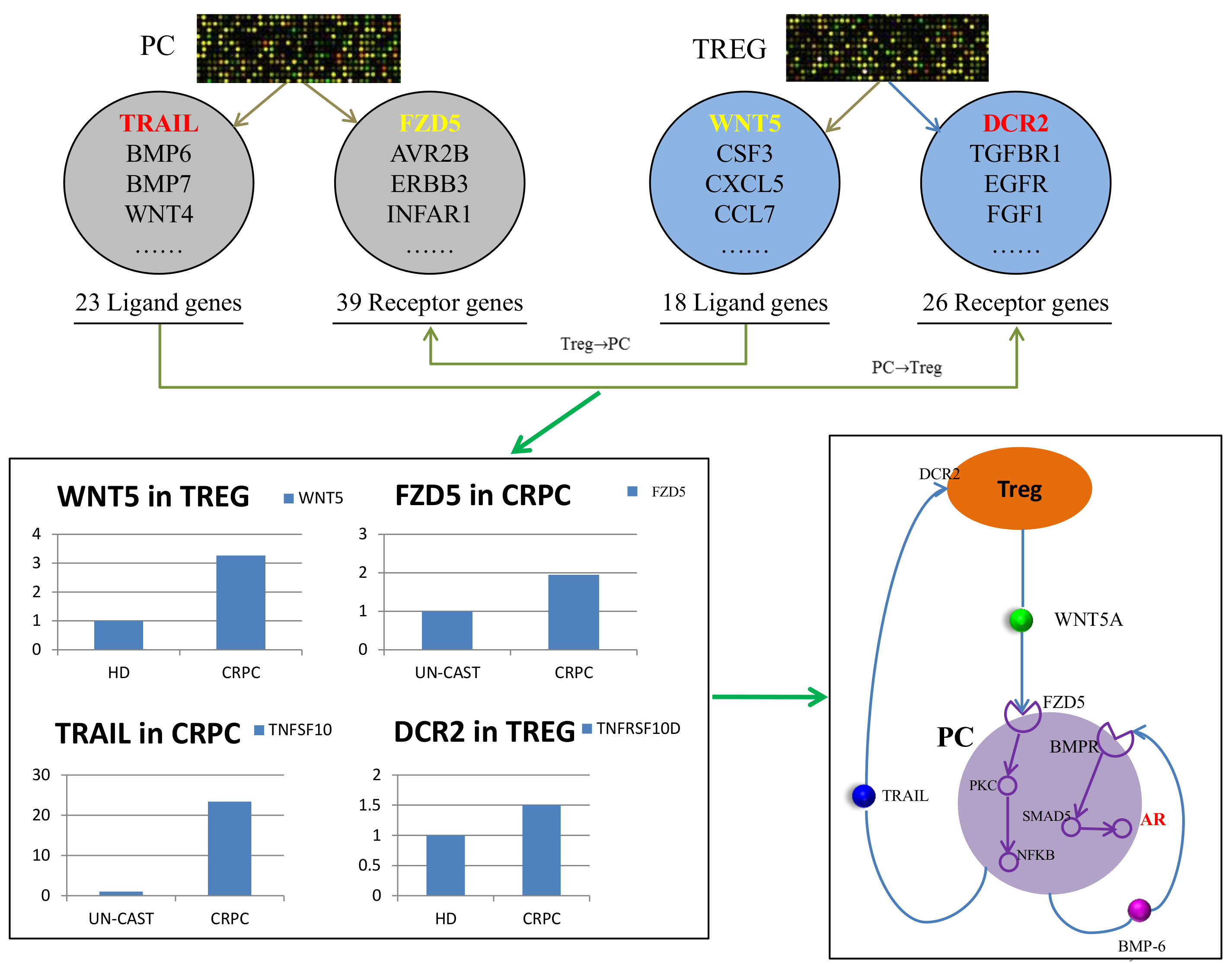

Supplement: S2 Fig — Twenty-three overexpressed ligand genes and 39 overexpressed receptor genes were identified from the dataset GSE46218, respectively. Eighteen overexpressed ligand genes and 26 receptor genes were identified from the dataset GSE38043. Based on the public ligand-receptor interaction database (iRefWeb) with a high confidence socore, a potential interaction pair was found: Treg→WNT5A→PC, and PC→TRAIL→Treg. (TIF) [file pcbi.1007344.s003.TIF]

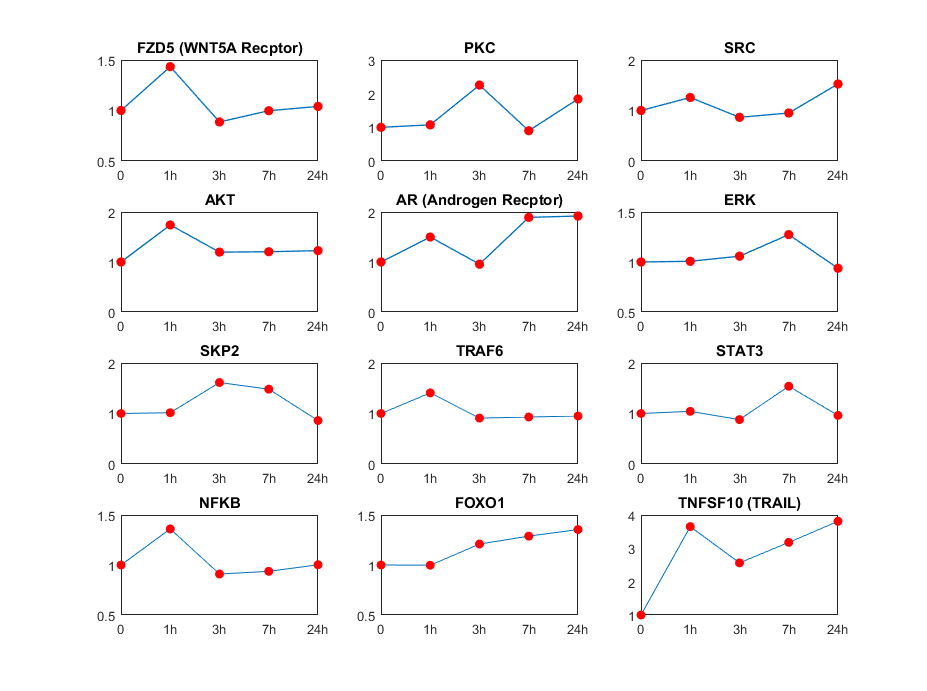

Supplement: S3 Fig — 22RV1 cells were treated with 250ng WNT5A and RNA samples collected at 1, 3, 7, and 24 hours. (TIF) [file pcbi.1007344.s004.tif]

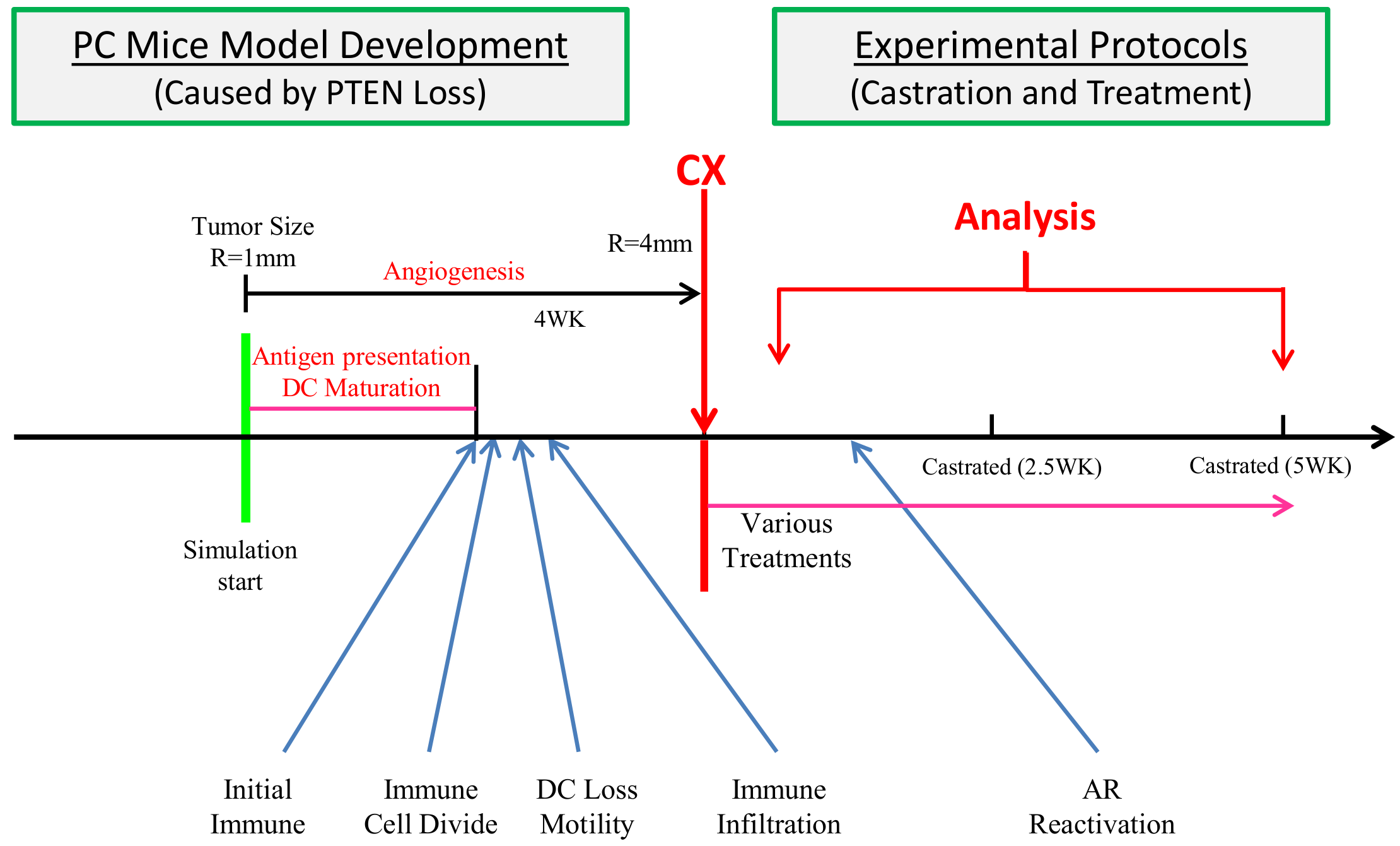

Supplement: S4 Fig — (TIF) [file pcbi.1007344.s005.TIF]

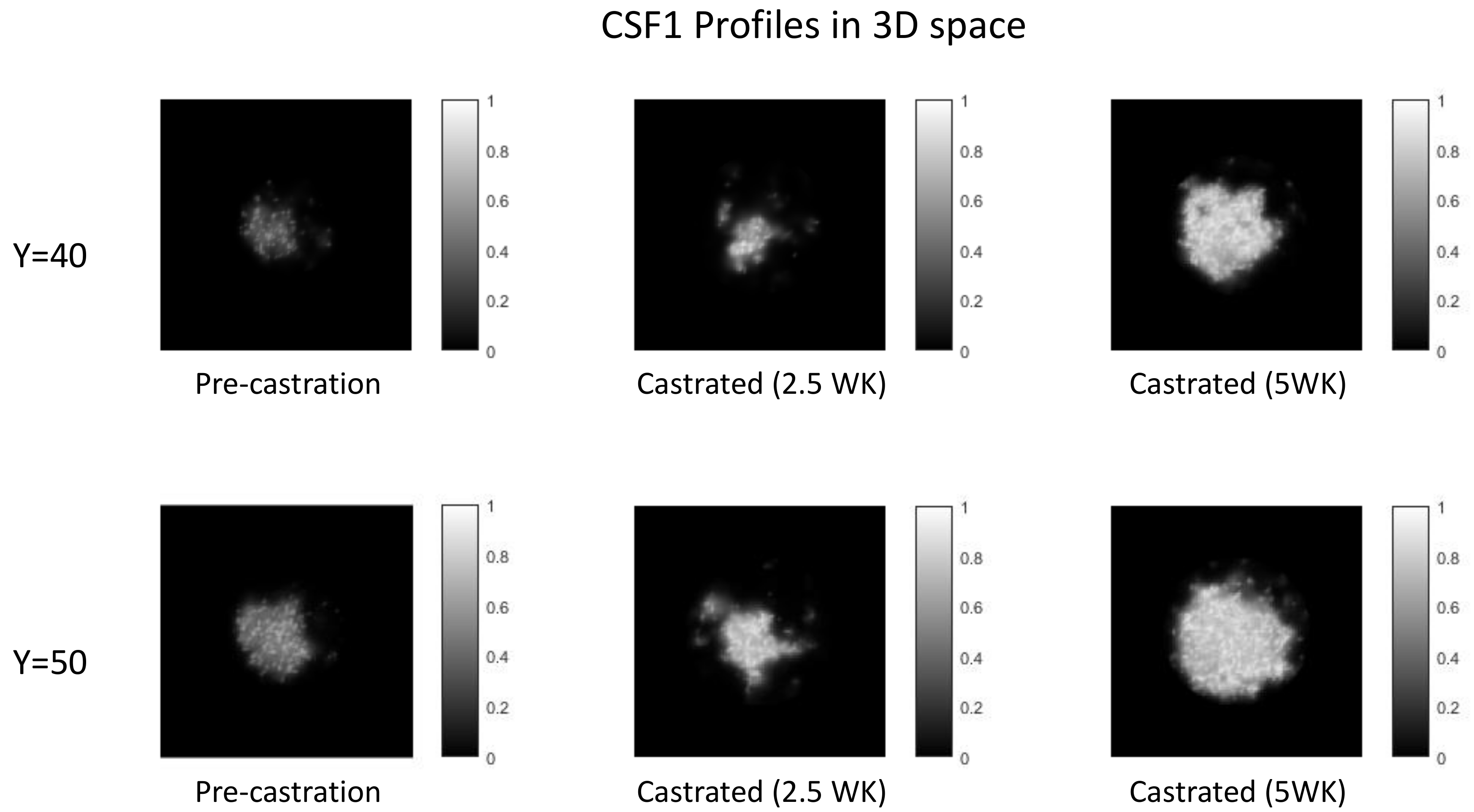

Supplement: S5 Fig — Two slices are presented: Y = 40, and Y = 50. Y is the Y axis (0≤Y≤100). (TIF) [file pcbi.1007344.s006.tif]

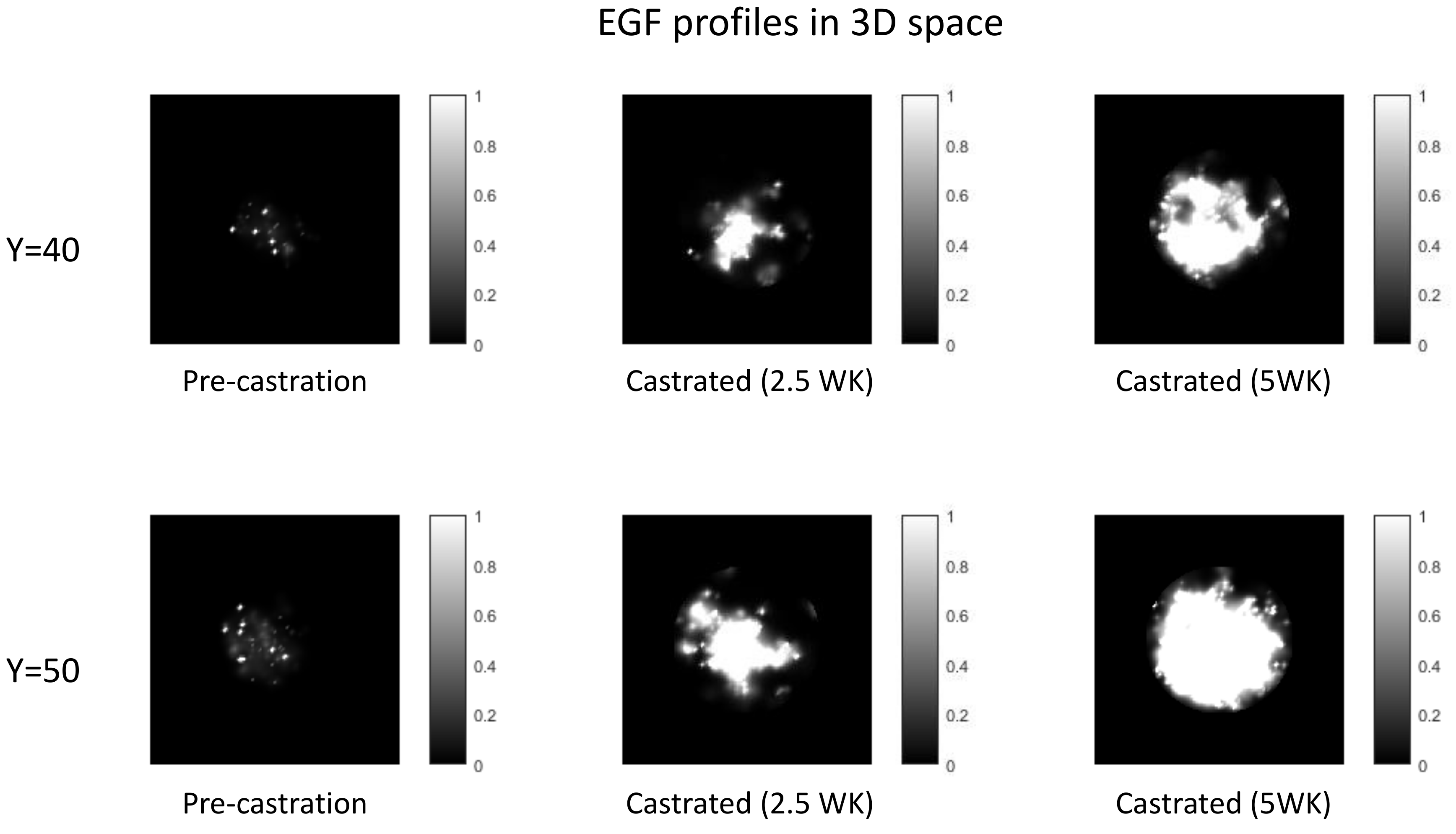

Supplement: S6 Fig — Two slices are presented: Y = 40, and Y = 50. Y is the Y axis (0≤Y≤100). (TIF) [file pcbi.1007344.s007.tif]

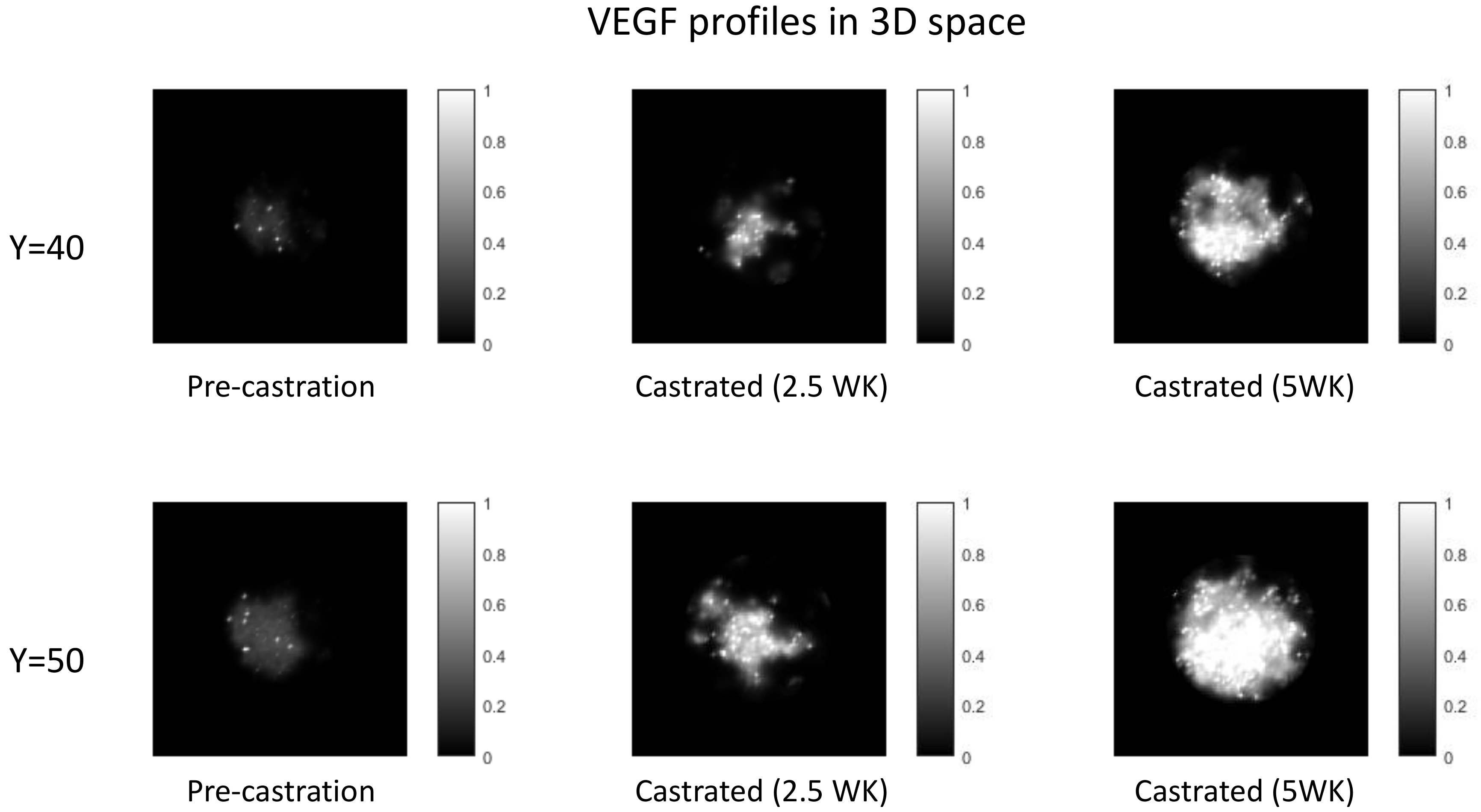

Supplement: S7 Fig — Two slices are presented: Y = 40, and Y = 50. Y is the Y axis (0≤Y≤100). (TIF) [file pcbi.1007344.s008.tif]

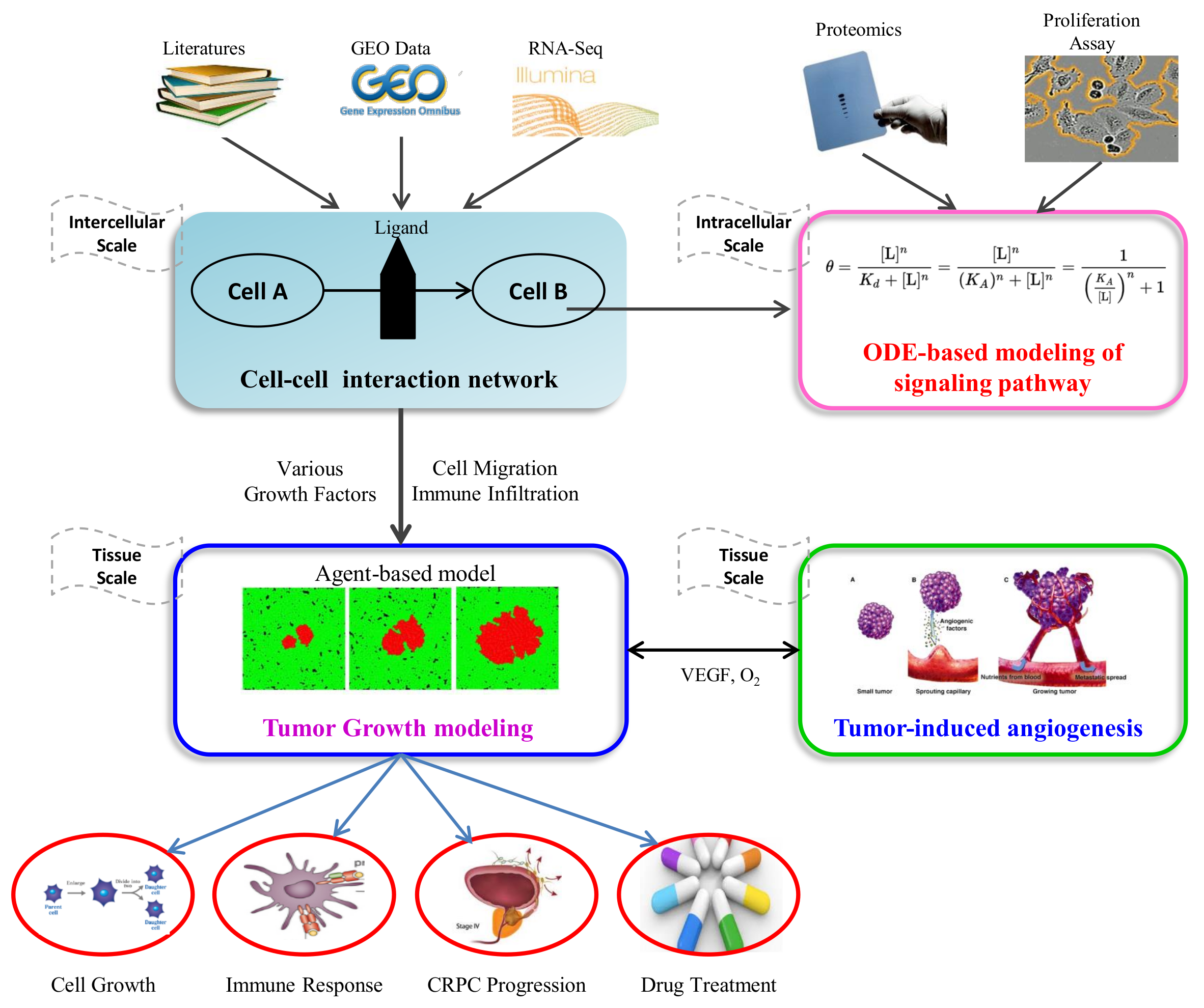

Supplement: S8 Fig — (TIF) [file pcbi.1007344.s009.TIF]

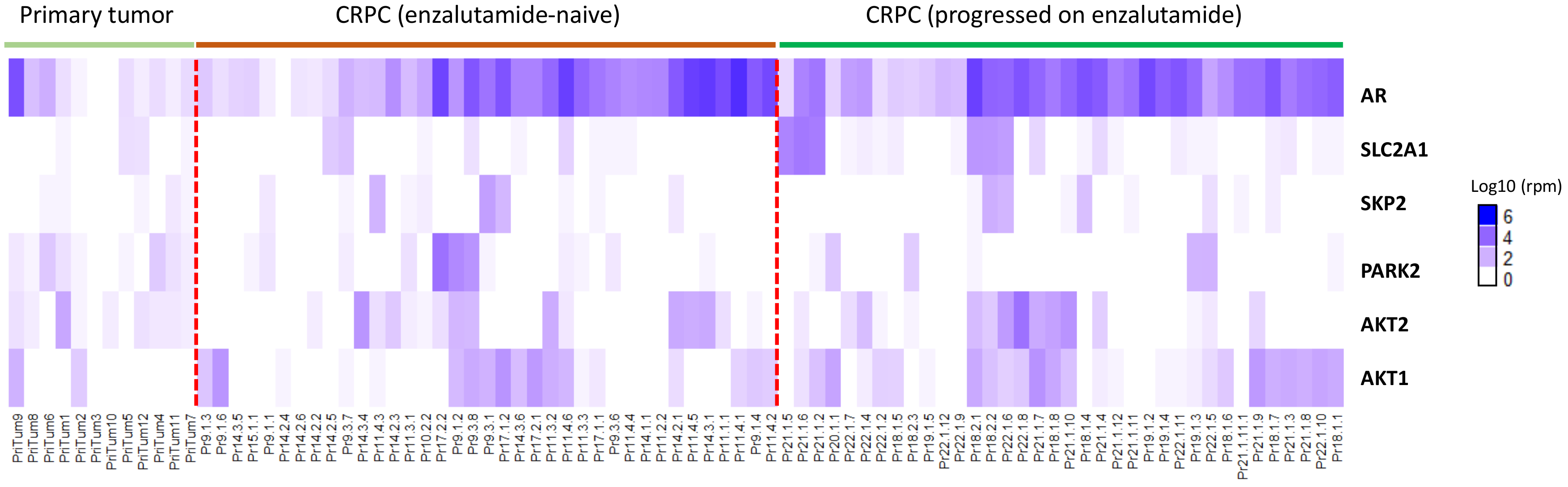

Supplement: S9 Fig — Primary tumor represents that the human tumor tissues were from PCa patients without treatment. CRPC (enzalutamide-naïve) indicates the human tumor tissues were from the PCa patients with CRPC occurrence who had not received enzalutamide. CRPC (progressed on enzalutamide represents the human tumor tissues were from the PCa patients who had received enzalutamide treatment after CRPC occurrence. (TIF) [file pcbi.1007344.s010.TIF]

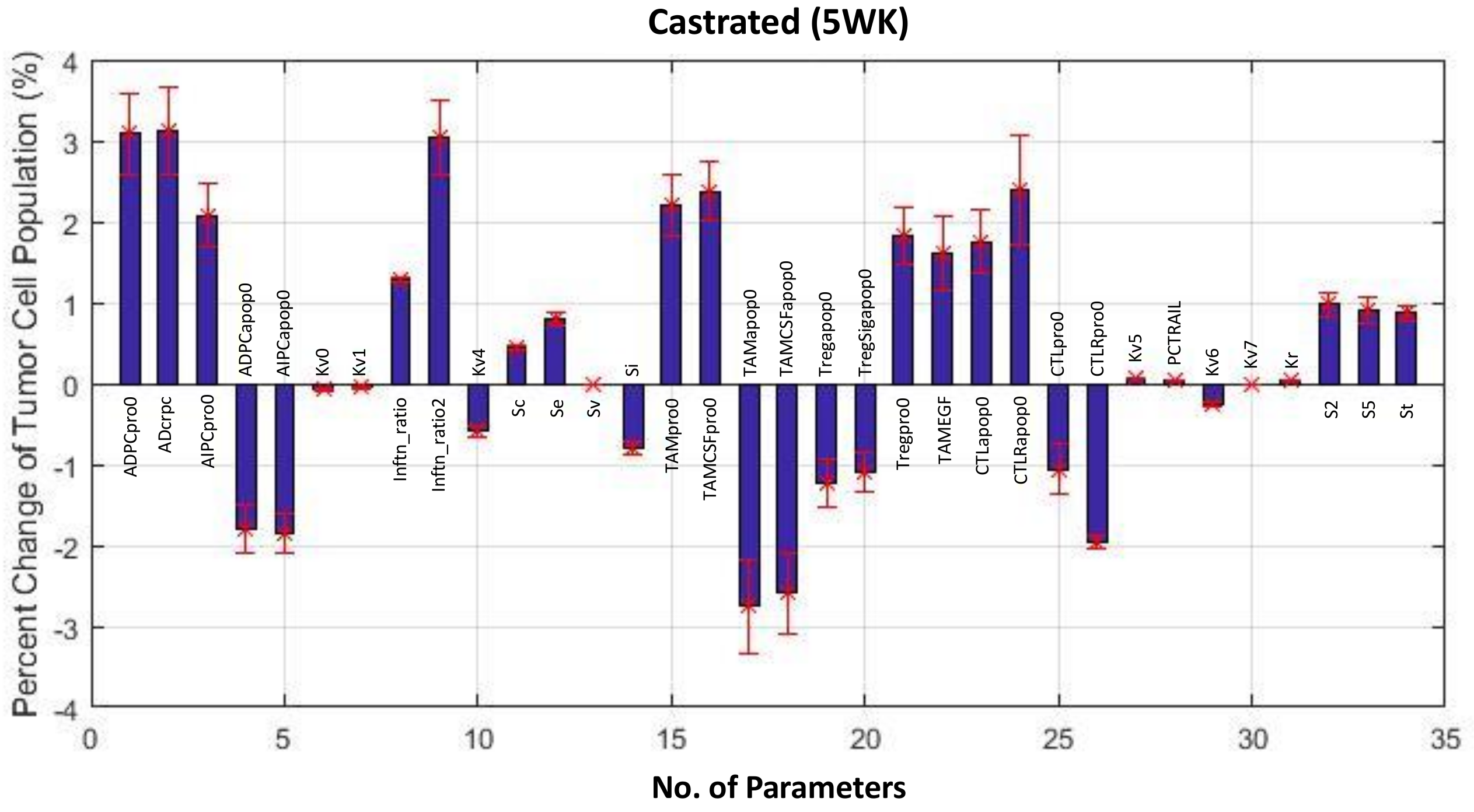

Supplement: S10 Fig — Sensitivity analysis was performed by measuring the impact of a small perturbation (5% increase) of individual 34 key parameters on the tumor cell (PC) population. (TIFF) [file pcbi.1007344.s011.TIFF]

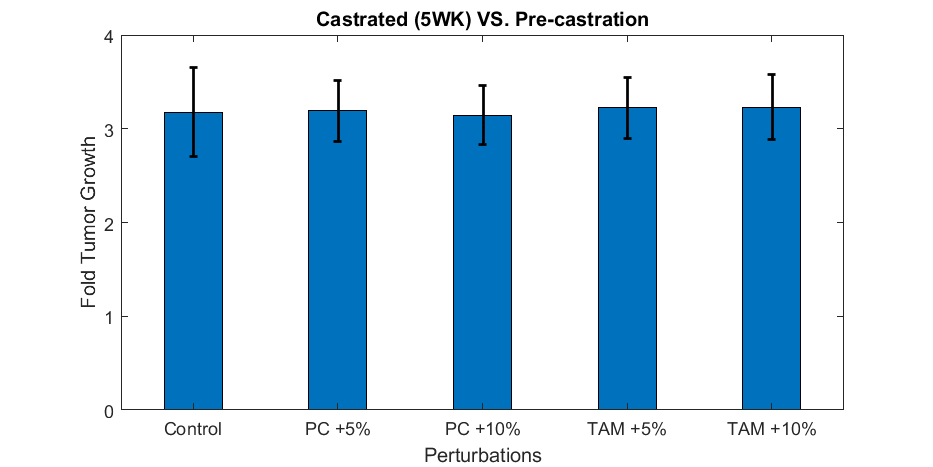

Supplement: S11 Fig — “Control” denotes the simulation without perturbation. The results are comparable with those in Fig 6L. The variability of the average value is in the range of -1.1139% to 1.7024%. (TIF) [file pcbi.1007344.s012.tif]

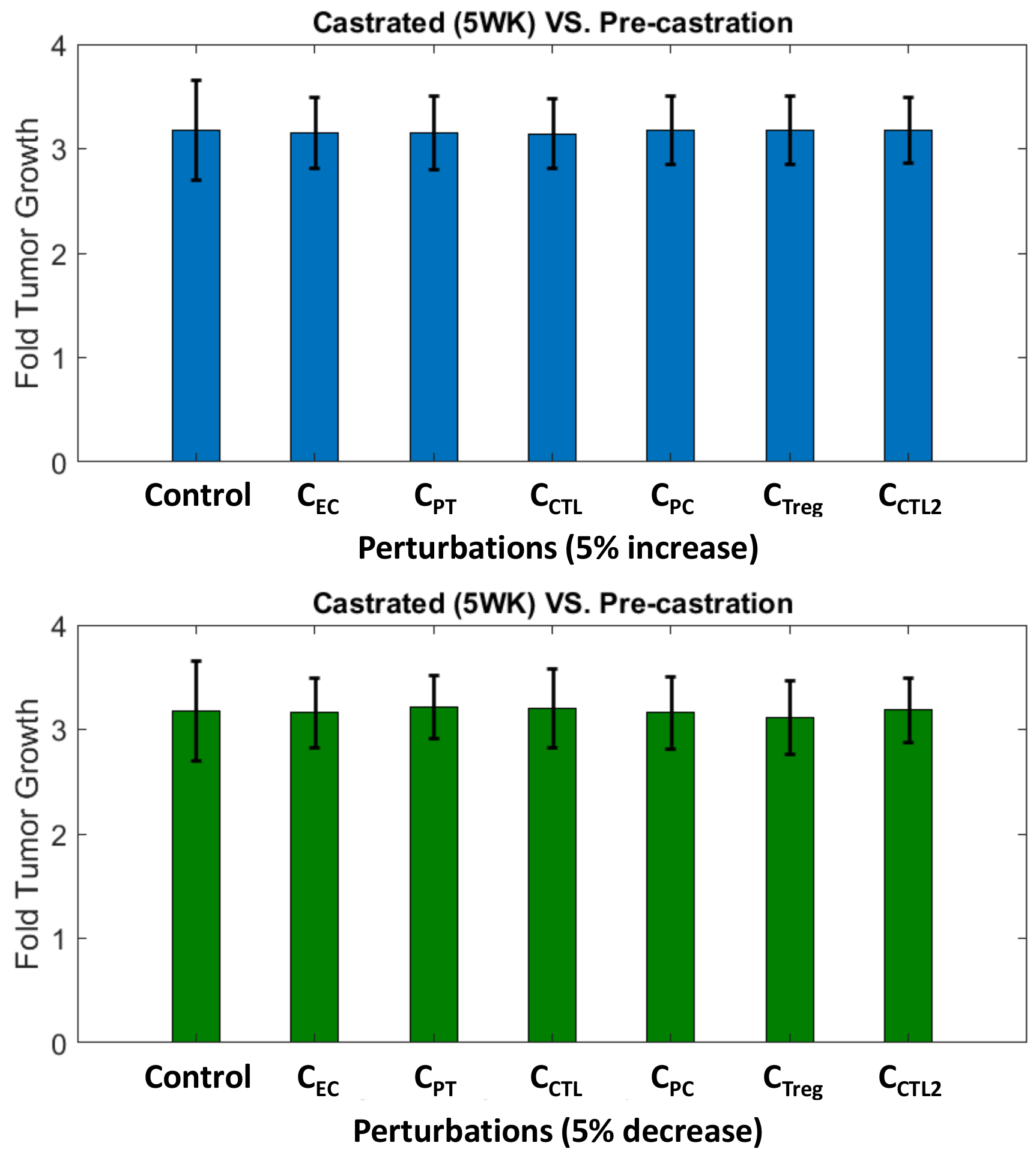

Supplement: S12 Fig — The fold change of tumor growth on day 35 (5 weeks) after castration was examined by a 5% increase or decrease in the individual cut-off value in the migration rules. “Control” denotes the simulation without perturbation. The results are comparable with those in Fig 6L. The variability of the average value for increase or decrease is in the range [-1.07%, 0.12%] and [-1.94%, 1.26%], respectively. (TIF) [file pcbi.1007344.s013.tif]

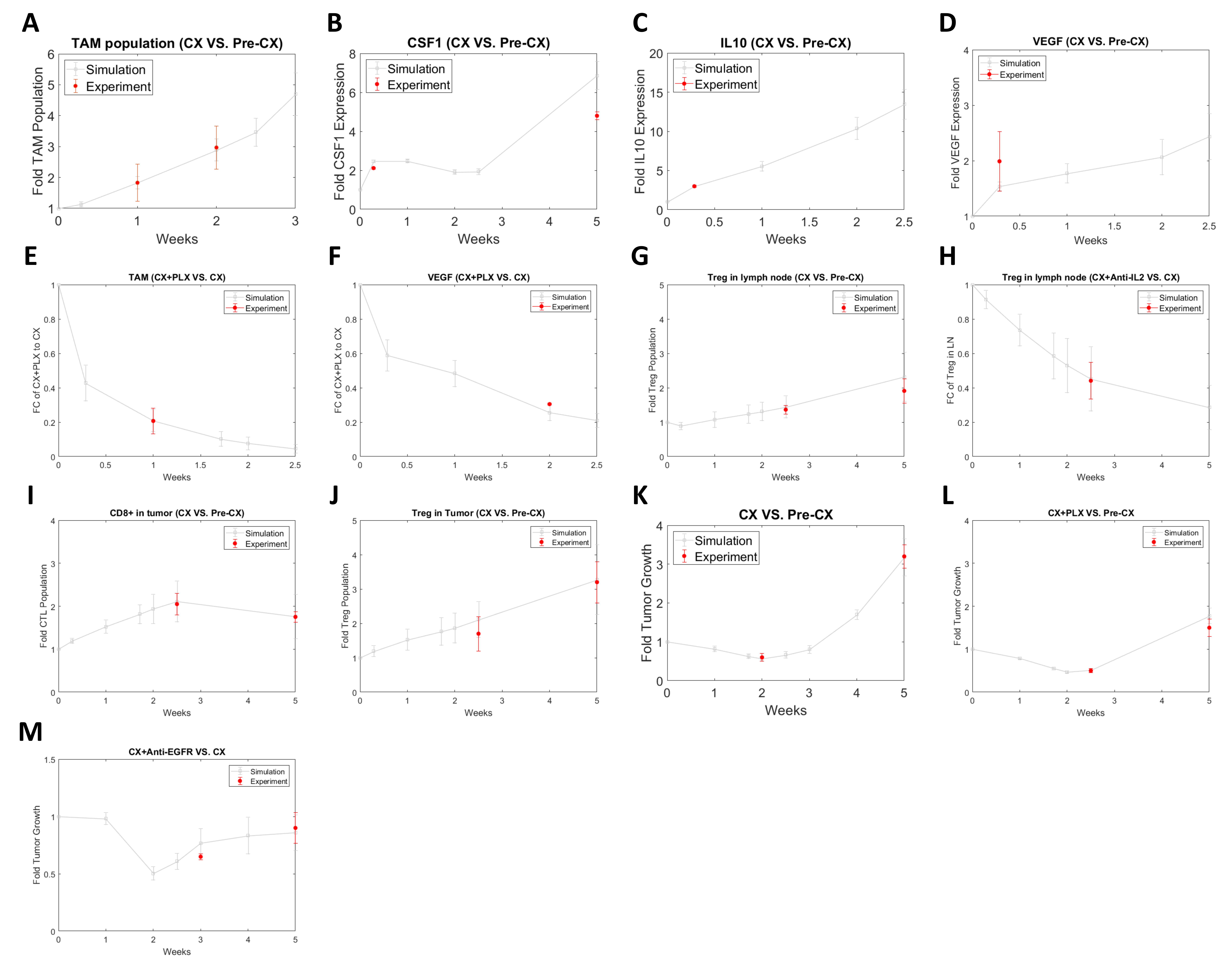

Supplement: S13 Fig — (A) The fold change of TAM population after castration relative to pre-castration. (B) The fold change of CSF1 expression after castration relative to pre-castration. (C-D) the fold change of IL10 and VEGF expressions after castration relative to pre-castration. (E-F) The fold change of TAM population and VEGF expression after the treatment with castration plus PLX comparted to castraton only. (G) The fold change of Treg population in lymph nodes after castration relative to pre-castration. (H) The fold change of Treg population in lymph nodes after the treatment with castration plus IL-2 neutralization compared to castration only. (I-J) The fold change of CD8+ and Treg population in tumor space after castration relative to pre-castration. (K) The fold change of tumor growth after castration relative to pre-castration. (L) The fold change of tumor growth after treatment with castration plus PLX compared to pre-castration. (M) The fold change of tumor growth after treatment with castration plus EGFR inhibitor to castration only. (TIF) [file pcbi.1007344.s014.TIF]

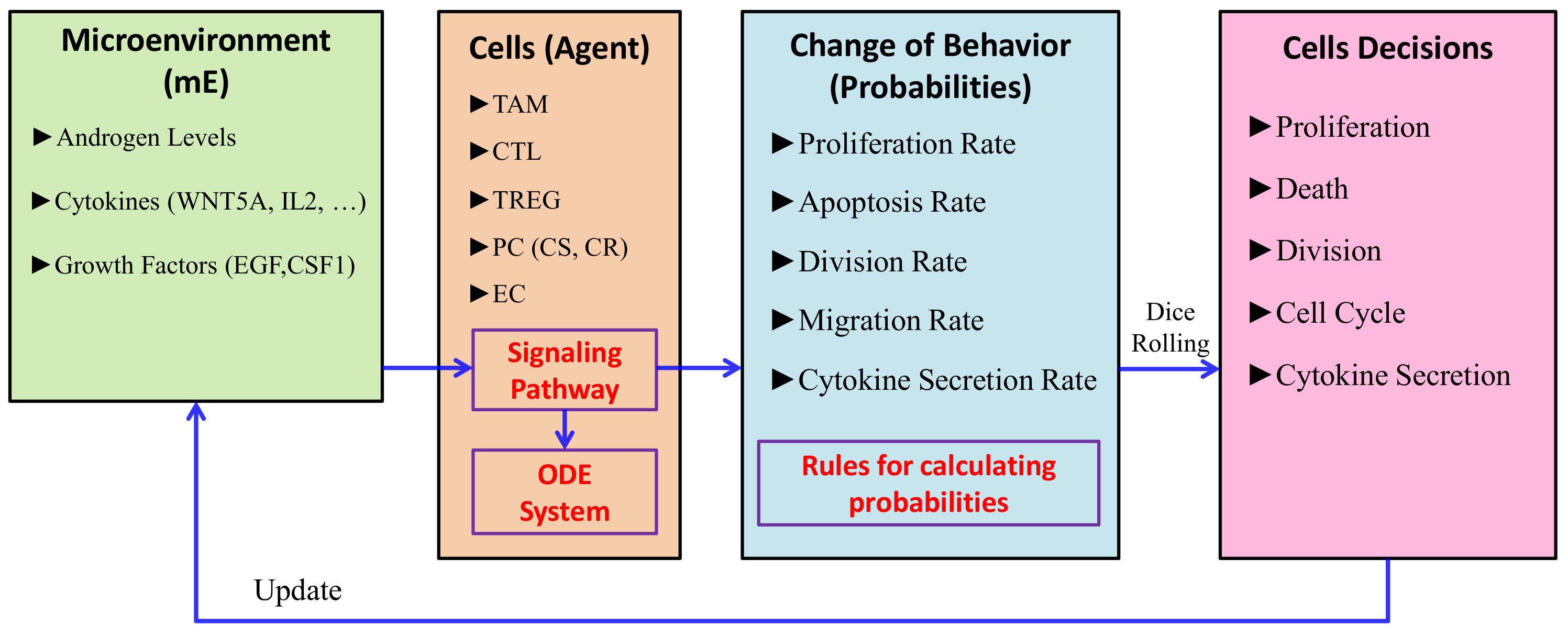

Supplement: S14 Fig — (TIF) [file pcbi.1007344.s015.TIF]

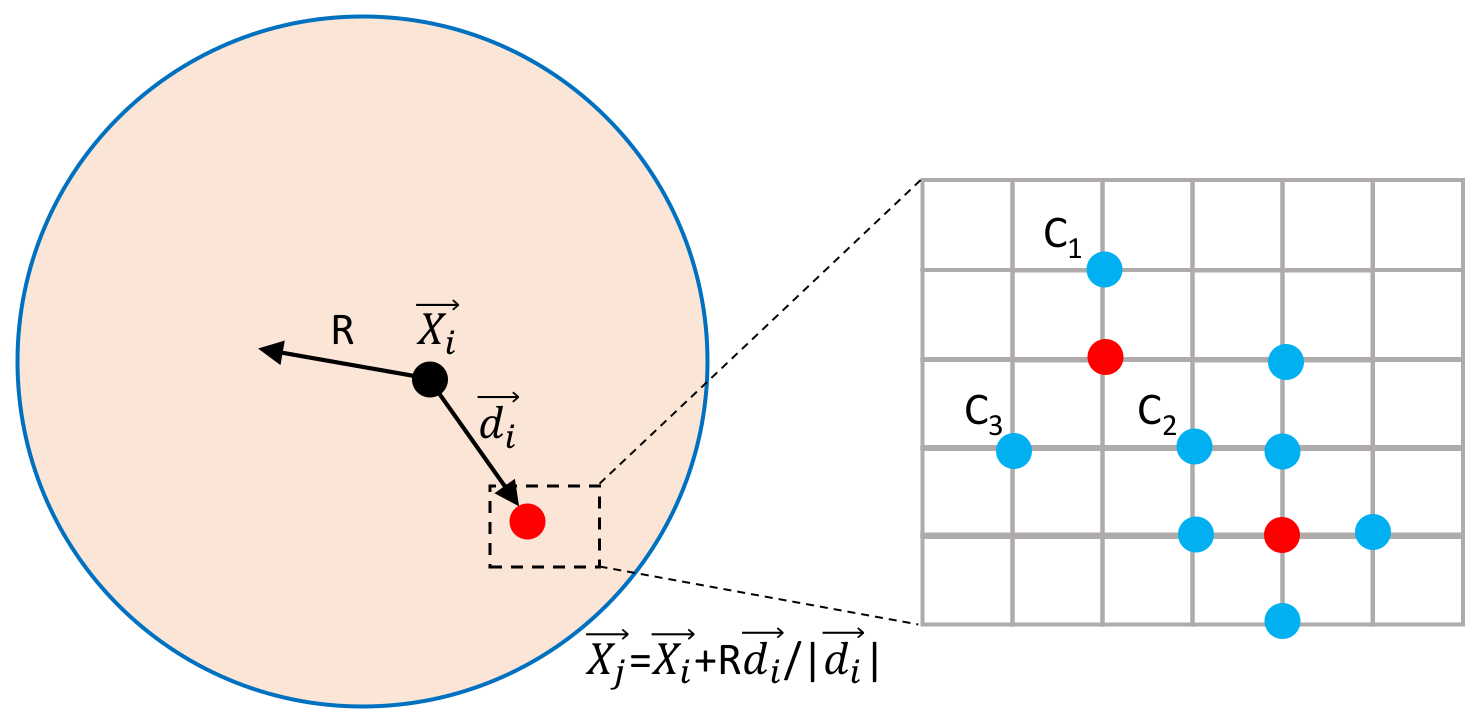

Supplement: S15 Fig — A cell at position Xi searches for the candidate locations within the distance R, and several empty positions (red dots) are identified. The probability (M) of a cell moving from Xi to Xj was determined by: 1) the moving offset (|di|); 2) the number of occupied cells (blue dots) around the new position; and 3) the type of the occupied cells. In our model, R equals 2 for migration, and 1 for proliferation. (TIF) [file pcbi.1007344.s016.TIF]

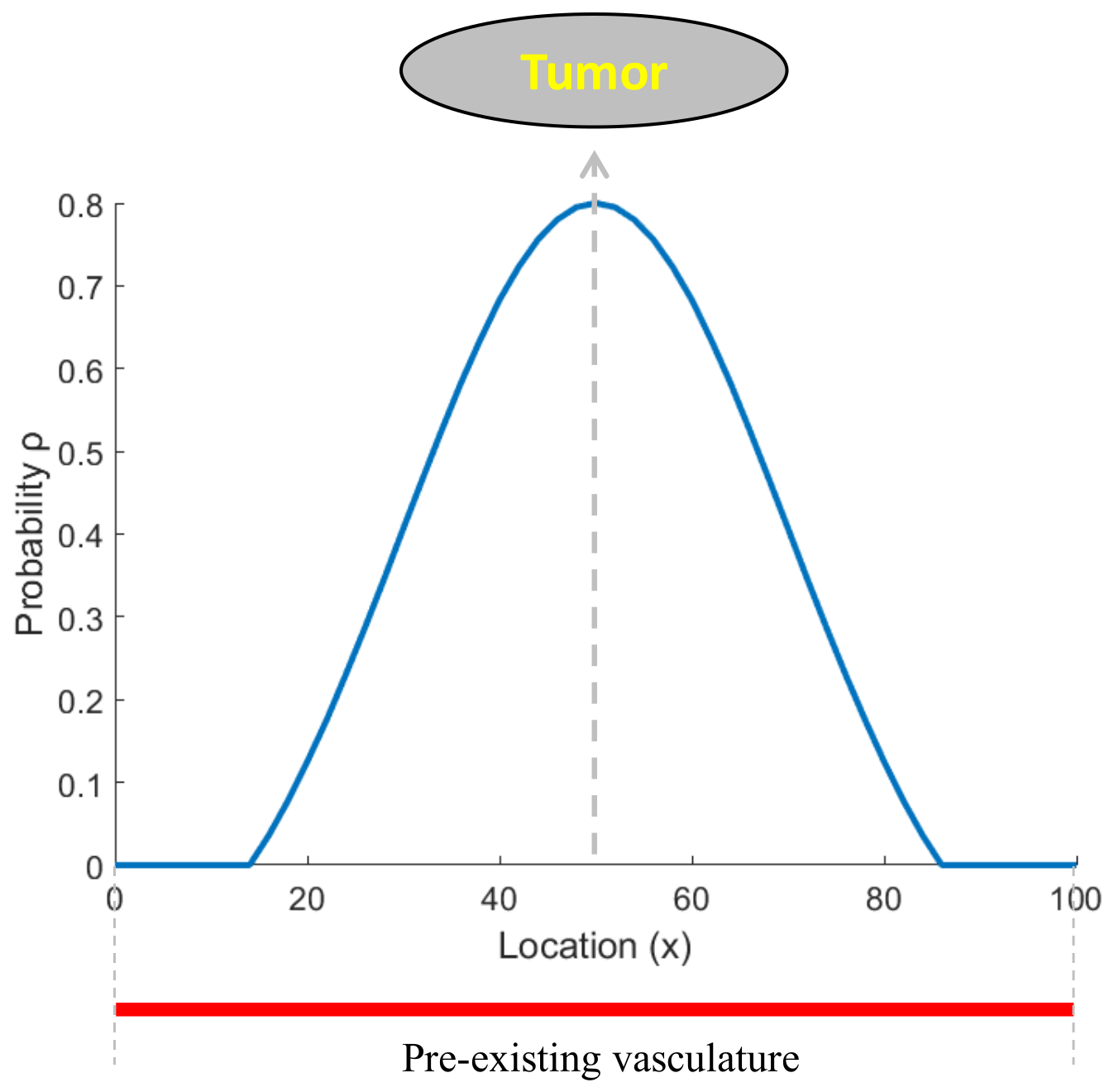

Supplement: S16 Fig — If 0≤x≤14 or 86≤x≤100, ρ = 0; otherwise, ρ follows a normal distribution (14<x<86). (TIFF) [file pcbi.1007344.s017.TIFF]

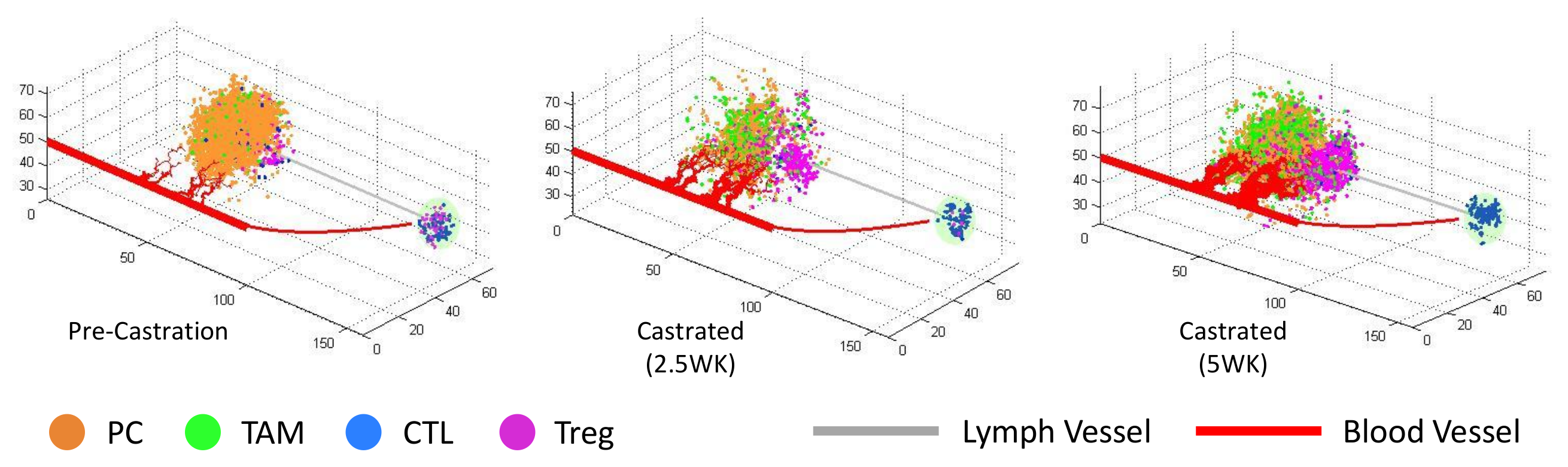

Supplement: S17 Fig — (TIF) [file pcbi.1007344.s018.TIF]
