# Supplementary material for: Systematically understanding the immunity leading to CRPC progression
Source: PLoS Comput Biol. 2019 Sep 10;15(9):e1007344. doi: 10.1371/journal.pcbi.1007344 (PMC6754164; doi:10.1371/journal.pcbi.1007344)
Supplement: S1 Table — (DOCX) [file pcbi.1007344.s019.docx]

**S1 Table.** Overexpressed ligands and receptors in PCs inferred from GSE46218 (P-value<0.05).

| **#** | **Gene Symbols** | **Entrez ID** | **Predicted Role** | **Confidence from iRefWeb** |
| --- | --- | --- | --- | --- |
| 1 | TNFSF10 (TRAIL) | 8743 | Ligand | high |
| 2 | BMP6 | 654 | Ligand | Low |
| 3 | BMP7 | 655 | Ligand | High |
| 4 | WNT4 | 54361 | Ligand | NA |
| 5 | WNT5A | 7474 | Ligand | High |
| 6 | NRG2 | 9542 | Ligand | High |
| 7 | DKK1 | 22943 | Ligand | High |
| 8 | GMFB | 2764 | Ligand | low |
| 9 | GRN | 2896 | Ligand | low |
| 10 | TNFSF15 | 9966 | Ligand | low |
| 11 | NMB | 4828 | Ligand | high |
| 12 | NOV | 4856 | Ligand | high |
| 13 | INHBB | 3625 | Ligand | high |
| 14 | PDGFC | 56034 | Ligand | high |
| 15 | PMCH | 5367 | Ligand | high |
| 16 | PRL | 5617 | Ligand | low |
| 17 | RLN1 | 6013 | Ligand | low |
| 18 | RLN2 | 6019 | Ligand | high |
| 19 | SECTM1 | 6398 | Ligand | low |
| 20 | CCL26 | 10344 | Ligand | low |
| 21 | CCL28 | 56477 | Ligand | high |
| 22 | CXCL1 | 6346 | Ligand | high |
| 23 | CXCL16 | 6360 | Ligand | high |
| 24 | FZD5 | 7855 | Receptor | low |
| 25 | ACVR2B | 93 | Receptor | low |
| 26 | ERBB3 | 2065 | Receptor | low |
| 27 | IFNAR1 | 3454 | Receptor | Low |
| 28 | FGFR3 | 2261 | Receptor | low |
| 29 | BRD8 | 10902 | Receptor | low |
| 30 | NCOA3 | 8202 | Receptor | low |
| 31 | BCAP31 | 10134 | Receptor | low |
| 32 | IL20RA | 53832 | Receptor | low |
| 33 | PTPRH | 5794 | Receptor | low |
| 34 | NOTCH3 | 4854 | Receptor | high |
| 35 | CD40 | 958 | Receptor | high |
| 36 | ITGA2 | 3673 | Receptor | low |
| 37 | GLP1R | 2740 | Receptor | low |
| 38 | RET | 5979 | Receptor | low |
| 39 | ITPR3 | 3710 | Receptor | low |
| 40 | TFRC | 7037 | Receptor | low |
| 41 | CD44 | 960 | Receptor | low |
| 42 | HPN | 3249 | Receptor | low |
| 43 | NR3C1 | 2908 | Receptor | high |
| 44 | PTPN12 | 5782 | Receptor | low |
| 45 | IFNGR2 | 3460 | Receptor | low |
| 46 | PTCH1 | 5727 | Receptor | NA |
| 47 | IL10RB | 3588 | Receptor | low |
| 48 | IL11RA | 3590 | Receptor | high |
| 49 | IL4R | 3566 | Receptor | high |
| 50 | IL21R | 50615 | Receptor | low |
| 51 | LRP2 | 4036 | Receptor | low |
| 52 | EPHA1 | 2041 | Receptor | low |
| 53 | EPHB3 | 2049 | Receptor | low |
| 54 | KIT | 3815 | Receptor | high |
| 55 | THRB | 7068 | Receptor | NA |
| 56 | NPR2 | 4882 | Receptor | low |
| 57 | NTSR1 | 4923 | Receptor | low |
| 58 | ITGB8 | 3696 | Receptor | low |
| 59 | LTBP1 | 4052 | Receptor | low |
| 60 | TNFRSF11A | 8792 | Receptor | low |
| 61 | MCC | 4163 | Receptor | low |
