# Supplementary material for: Systematically understanding the immunity leading to CRPC progression
Source: PLoS Comput Biol. 2019 Sep 10;15(9):e1007344. doi: 10.1371/journal.pcbi.1007344 (PMC6754164; doi:10.1371/journal.pcbi.1007344)
Supplement: S2 Table — (DOCX) [file pcbi.1007344.s020.docx]

**S2 Table.** Overexpressed ligands and receptors in Tregs inferred from GSE38043 (P-value<0.05).

| **#** | **Gene Symbols** | **Entrez ID** | **Predicted Role** | **Confidence from iRefWeb** |
| --- | --- | --- | --- | --- |
| 1 | WNT5A | 7474 | Ligand | high |
| 2 | CSF3 | 1440 | Ligand | low |
| 3 | CCL7 | 6354 | Ligand | high |
| 4 | CORT | 1325 | Ligand | high |
| 5 | CXCL5 | 6374 | Ligand | high |
| 6 | FASLG | 356 | Ligand | high |
| 7 | FGF18 | 8817 | Ligand | low |
| 8 | GNRH1 | 2796 | Ligand | low |
| 9 | IL17A | 3605 | Ligand | high |
| 10 | IL1A | 3552 | Ligand | high |
| 11 | IL1B | 3553 | Ligand | high |
| 12 | IL37 | 27178 | Ligand | low |
| 13 | MST1 | 4485 | Ligand | NA |
| 14 | OSM | 5008 | Ligand | high |
| 15 | PF4 | 5196 | Ligand | low |
| 16 | PGF | 5228 | Ligand | low |
| 17 | TNFSF14 | 8740 | Ligand | high |
| 18 | TYMP | 1890 | Ligand | low |
| 19 | TNFRSF10D (DCR2) | 8793 | Receptor | high |
| 20 | TGFBR1 | 7046 | Receptor | high |
| 21 | EGFR | 1956 | Receptor | high |
| 22 | FGFR1 | 2260 | Receptor | high |
| 23 | ADIPOR2 | 79602 | Receptor | low |
| 24 | ACVR2A | 92 | Receptor | high |
| 25 | ZFYVE9 | 9372 | Receptor | low |
| 26 | ERBB4 | 2066 | Receptor | low |
| 27 | CD40 | 958 | Receptor | high |
| 28 | MED17 | 9440 | Receptor | low |
| 29 | CSF3R | 1441 | Receptor | low |
| 30 | GOSR1 | 9527 | Receptor | low |
| 31 | F2R | 2149 | Receptor | high |
| 32 | THRAP3 | 9967 | Receptor | low |
| 33 | GLP1R | 2740 | Receptor | low |
| 34 | ACVR1B | 91 | Receptor | low |
| 35 | CD44 | 960 | Receptor | low |
| 36 | IL12RB1 | 3594 | Receptor | high |
| 37 | IL18RAP | 8807 | Receptor | high |
| 38 | IL1RL1 | 9173 | Receptor | high |
| 39 | EPHA1 | 2041 | Receptor | low |
| 40 | ROR1 | 4919 | Receptor | low |
| 41 | FLT1 | 2321 | Receptor | high |
| 42 | MC3R | 4159 | Receptor | low |
| 43 | ITGB8 | 3696 | Receptor | low |
| 44 | TNFRSF9 | 3604 | Receptor | high |
