# Supplementary material for: Systematically understanding the immunity leading to CRPC progression
Source: PLoS Comput Biol. 2019 Sep 10;15(9):e1007344. doi: 10.1371/journal.pcbi.1007344 (PMC6754164; doi:10.1371/journal.pcbi.1007344)
Supplement: S3 Table — (DOCX) [file pcbi.1007344.s021.docx]

**S3 Table.** Enriched pathways associated with significantly expressed genes after WNT5A treatment (Top 10 enriched pathways obtained from KEGG, P-value<0.05).

| **Ko_id** | **Enriched Pathways** | **No. of genes** |
| --- | --- | --- |
| ko05200 | Pathways in cancer | 121 |
| ko04151 | PI3K-AKT signaling pathway | 81 |
| ko04014 | Ras signaling pathway | 71 |
| ko04010 | MAPK signaling pathway | 71 |
| ko04060 | Cytokine-cytokine receptor interaction | 61 |
| ko04510 | Focal adhesion | 57 |
| ko04630 | Jak-STAT signaling pathway | 43 |
| ko04150 | mTOR signaling pathway | 30 |
| ko05215 | Prostate cancer | 29 |
| ko04310 | Wnt signaling pathway | 27 |
