# Supplementary material for: Systematically understanding the immunity leading to CRPC progression
Source: PLoS Comput Biol. 2019 Sep 10;15(9):e1007344. doi: 10.1371/journal.pcbi.1007344 (PMC6754164; doi:10.1371/journal.pcbi.1007344)
Supplement: S4 Table — (DOCX) [file pcbi.1007344.s022.docx]

**S4 Table.** Enriched genes of TAM and PCa cells identified from our RNA-seq data (FC>1.3).

|  | **Over-expressed Ligand Genes** | **Over-expressed Receptor Genes** |
| --- | --- | --- |
| **LnCAP W/WO TAM** | SPP1, TNFSF10, GCG, CNTF, TNFSF15,  TTR, PMCH, CMTM8, NMB, VEGFA,ANGPT2 | ITGB2, PTPN14, BMPR2, NOTCH1, SDC4, AR |
| **22RV1 W/WO TAM** | TNFSF10, SPP1, TGFB1, CHGB, CSF1, ADM, VEGFA, TNFSF15, ADCYAP1, DKK1, JAG2, MIF, UCN | AMHR2, EPHB3, RAMP2, CRLF1, CXCR4, VTN, CCR10, TNFRSF12A, EGFR, DPP4, MED16, FZD9 |
| **TAM W/WO LnCAP** | HBEGF, CCL20, CCL21, SPP1, IL10, TNF, HGF, PDGFA, TNFSF10, CCL4, CCL5, CD70, CXCL1, EBI3, IL18, IL1B, IL23A, LTB, NMB, OSM, POMC, PRL, SECTM1, TNFSF13, TNFSF14, TNFSF9, TYMP | CSF1R, IFNGR2, IL10RB, TNFRSF10A, ITGB5, CD40, FZD6, CXCR2, RAMP1, CCR1, CCR2, PHB2, HLA-B, CSF3R, FLT3, MLNR, TFRC, IL4R, MED30, TNFRSF10C, EB13, TNFRSF14, TNFRSF1B, ADORA2A, PLAUR, OPRL1, RXFP1, TNFRSF25, TNFRSF9, SFRP1 |
| **TAM W/WO 22RV1** | CCL20, SPP1, CCL28, HGF, TNFSF10, IL10, TNF, VEGFA, ADM, BMP8B, CCL21, CCL3L3, CCL4, CD70, CXCL1, CXCL16, EBI3, GRN, HBEGF, IL16, IL18, IL1B, MIF, NMB, OSM, PDGFA, PRL, TNFSF14, TNFSF9, TYMP, UCN | CXCR4, NOTCH1, ITGB5, ITGA5, IFNGR2, IL10RB, CSF1R, IFNGR1, ITGAV, CCR2, IL6R, PHB, CSF2RA, CSF3R, CX3CR1, CXCR2, ITGAL, FGFRL1, FLT3, MLNR, HLA-DQB1, THRA, IL11RA, EBI3, IL4R, IL13RA1, EFEMP2, IL17RA, IL1RAP, ADRB2, IL21R, MED30, PTPN14, TNFRSF1B, TNFRSF10D, CD74, ADORA2A, PLAUR, OPRL1, RXFP1, TGFBR1, TNFRSF10A, TNFRSF12A, TRAF1, SFRP1, FZD7 |
