# Supplementary material for: Systematically understanding the immunity leading to CRPC progression
Source: PLoS Comput Biol. 2019 Sep 10;15(9):e1007344. doi: 10.1371/journal.pcbi.1007344 (PMC6754164; doi:10.1371/journal.pcbi.1007344)
Supplement: S5 Table — (DOCX) [file pcbi.1007344.s023.docx]

**S5 Table.** The parameters in ODE system optimized by GA algorithm.

| **No.** | **Parameters** | **Estimated value** | **Associated with ODE Functions** |
| --- | --- | --- | --- |
| 1 | K_1_ | 0.9517 | Eq. (1) |
| 2 | H_1_ | 0.7881 | Eq. (1) |
| 3 | d_1_ | 0.1455 | Eq. (1) |
| 4 | K_2_ | 0.8705 | Eq. (2) |
| 5 | H_2_ | 0.6913 | Eq. (2) |
| 6 | d_2_ | 04653 | Eq. (2) |
| 7 | K_3_ | 0.9960 | Eq. (3) |
| 8 | H_3_ | 0.1018 | Eq. (3) |
| 9 | d_3_ | 0.8413 | Eq. (3) |
| 10 | K_4_ | 0.9934 | Eq. (4) |
| 11 | H_4_ | 0.2854 | Eq. (4) |
| 12 | K_5_ | 0.0651 | Eq. (4) |
| 13 | H_5_ | 0.0355 | Eq. (4) |
| 14 | d_4_ | 0.0571 | Eq. (4) |
| 15 | K_6_ | 0.3594 | Eq. (5) |
| 16 | H_6_ | 0.8551 | Eq. (5) |
| 17 | d_5_ | 0.7088 | Eq. (5) |
