# Supplementary material for: Systematically understanding the immunity leading to CRPC progression
Source: PLoS Comput Biol. 2019 Sep 10;15(9):e1007344. doi: 10.1371/journal.pcbi.1007344 (PMC6754164; doi:10.1371/journal.pcbi.1007344)
Supplement: S6 Table — (DOCX) [file pcbi.1007344.s024.docx]

**S6 Table.** The inferred parameters for agent-based model in HMSM.

| **Symbol** | **Variable** | **Initial value** | **Reference** |
| --- | --- | --- | --- |
| ACcrpc | The local androgen level in CRPC phase. | 0.68 | * |
| ADPCpro0 | Proliferation rate of PCs via androgen dependent pathways. | 0.0385 | * |
| AIPCpro0 | Proliferation rate of PCs via androgen independent pathways. | 0.011 | * |
| ADPCapop0 | Apoptosis rate of PCs via androgen dependent pathways. | 0.011 | * |
| AIPCapop0 | Apoptosis rate of PCs via androgen independent pathways. | 0.012 | * |
| TAMpro0 | Initial proliferation rate of TAM cells. | 0.010 | * |
| TAMCSFpro0 | Proliferation rate of TAM cells induced by activated CSF1R. | 0.0175 | * |
| TAMapop0 | Initial apoptosis rate of TAM cells. | 0.0147 | * |
| TAMCSFapop0 | The apoptosis rate of TAM cells induced by CSF1R inhibitor. | 0.0138 | * |
| $K_{v0}$ | Coefficient of Hill Function in Eq. (1) described above. | 1.065 | * |
| $K_{v1}$ | Coefficient of Hill Function in Eq. (8) described above. | 0.5 | * |
| $K_{v4}$ | Coefficient of Hill Function in Eq. (2) described above. | 2.7 | * |
| Sc | Secretion rate of CSF1 after Castration. | 2 | * |
| Se | Secretion rate of EGF after Castration. | 4 | * |
| Sv | Secretion rate of VEGF after Castration. | 25 | * |
| Si | Secretion rate of IL10 after Castration. | 3 | * |
| Tregapop0 | Initial apoptosis rate of Tregs. | 0.011 | * |
| TregSigapop0 | Apoptosis rate induced by anti-IL2. | 0.0049 | * |
| Tregpro0 | Initial proliferation rate of Tregs. | 0.01 | (1) |
| CTLapop0 | Initial apoptosis rate of CTLs. | 0.011 | (1) |
| CTLRapop0 | Apoptosis rate of CTLS promoted by Tregs population. | 0.02 | * |
| CTLpro0 | Initial proliferation rate of CTLs. | 0.012 | * |
| CTLRpro0 | Proliferation rate of CTLS inhibited by Tregs population. | 0.05 | * |
| $K_{v5}$ | Coefficient of Hill Function in Eq. (4) described above. | 0.6 | * |
| $K_{v6}$ | Coefficient of Hill Function in Eq. (3) described above. | 1.3 | * |
| $K_{v7}$ | Coefficient of Hill Function in Eq. (3) described above. | 0.3 | * |
| *Kr* | Coefficient of Hill Function in Eq. (5) described above. | 0.5 | * |
| S2 | Secretion rate of IL2 after Castration. | 4 | * |
| S5 | Secretion rate of WNT5A after Castration. | 4 | * |
| St | Secretion rate of TRAIL after Castration. | 4 | * |
| ECapop0 | Apoptosis rate of ECs. | 0.0000001 | * |
| ECpro0 | Proliferation rate of ECs. | 0.157 | * |
| Inftn_ratio | Infiltration rate of Treg before Castration. | 0.01 | * |
| Inftn_ratio2 | Infiltration rate of Treg after Castration. | 0.013 | * |
| Inftn_ratioa | Infiltration rate of CTL before Castration. | 0.006 | * |
| Inftn_ratiob | Infiltration rate of CTL after Castration. | 0.0025 | * |
| MAXANTI | Maximal concentration level of PSA in the simulated system. | 20 | (2) |
| MINANTI | Minimal concentration level of PSA. | 0 | * |
| PCANTI | Unit concentration of PSA secreted from each dead PC. | 9 | * |
| MAXDHT | Maximal concentration level of DHT (androgen). | 20 | (2, 3) |
| MINDHT | Minimal concentration level of DHT (androgen). | 2 | * |
| PCDHT | Unit concentration of DHT generated in PCs after AR reactivation. | 0.5 | * |
| MAXCSF | Maximal concentration level of CSF1. | 5 | * |
| MINCSF | Minimal concentration level of CSF1. | 0.001 | * |
| PCCSF | Unit concentration of CSF1 secreted from each PC. | 0.6 | * |
| MAXIL2 | Maximal concentration level of IL2. | 1 | * |
| MINIL2 | Minimal concentration level of IL2. | 0 | * |
| CTLIL2 | Unit concentration of IL2 secreted from each PC. | 0.2 | * |
| MAXEGF | Maximal concentration level of EGF. | 20 | * |
| MINEGF | Minimal concentration level of EGF. | 0 | * |
| TAMEGF | Unit concentration of EGF secreted from each TAM. | 4 | * |
| MAXWNT | Maximal concentration level of WNT5A. | 1 | * |
| MINWNT | Minimal concentration level of WNT5A. | 0 | * |
| TREGWNT5A | Unit concentration of WNT5A secreted from each Treg. | 0.2 | * |
| MAXTRAIL | Maximal concentration level of TRAIL. | 1 | * |
| MINTRAIL | Minimal concentration level of TRAIL. | 0 | * |
| PCTRAIL | Unit concentration of TRAIL secreted from each PC. | 0.2 | * |
| MAXIL10 | Maximal concentration level of IL10. | 3.0 | * |
| MINIL10 | Minimal concentration level of IL10. | 0.001 | * |
| TAMIL10 | Unit concentration of IL10 secreted from each TAM. | 0.5 | * |
| MAXVEGF | Maximal concentration level of VEGF. | 8.5 | (4) |
| MINVEGF | Minimal concentration level of VEGF. | 0.001 | (4) |
| TAMVEGF | Unit concentration of VEGF secreted from each TAM. | 0.35 | (4) |
| PCVEGF | Unit concentration of VEGF secreted from each PC. | 0.35 | (4) |
| Lamada_GF | Diffusion constant of growth factor. | 0.52 | (5) |
| Lamada_VEGF | Diffusion constant of VEGF. | 0.835 | (6) |
| DEG | Degradation rate of cytokines. | 0.05 | (1) |

**Supplementary Reference**

1. Ji Z, Su J, Wu D, Peng H, Zhao W, Nlong Zhao B, et al. Predicting the impact of combined therapies on myeloma cell growth using a hybrid multi-scale agent-based model. Oncotarget. 2017;8(5):7647-65.

2. Baez J, Kuang Y. Mathematical Models of Androgen Resistance in Prostate Cancer Patients under Intermittent Androgen Suppression Therapy. Applied Sciences-Basel. 2016;6(11).

3. Bruchovsky N, Klotz L, Crook J, Malone S, Ludgate C, Morris WJ, et al. Final results of the Canadian prospective phase II trial of intermittent androgen suppression for men in biochemical recurrence after radiotherapy for locally advanced prostate cancer: clinical parameters. Cancer. 2006;107(2):389-95.

4. Sun XQ, Zhang L, Tan H, Bao JG, Strouthos C, Zhou XB. Multi-scale agent-based brain cancer modeling and prediction of TKI treatment response: Incorporating EGFR signaling pathway and angiogenesis. Bmc Bioinformatics. 2012;13.

5. Su J, Zhang L, Zhang W, Choi DS, Wen J, Jiang B, et al. Targeting the biophysical properties of the myeloma initiating cell niches: a pharmaceutical synergism analysis using multi-scale agent-based modeling. PLoS One. 2014;9(1):e85059.

6. Wang J, Zhang L, Jing C, Ye G, Wu H, Miao H, et al. Multi-scale agent-based modeling on melanoma and its related angiogenesis analysis. Theor Biol Med Model. 2013;10:41.
