# Supplementary material for: Systematically understanding the immunity leading to CRPC progression
Source: PLoS Comput Biol. 2019 Sep 10;15(9):e1007344. doi: 10.1371/journal.pcbi.1007344 (PMC6754164; doi:10.1371/journal.pcbi.1007344)
Supplement: S7 Table — (DOCX) [file pcbi.1007344.s025.docx]

**S7 Table.** The sequences of the paired sense and antisense primers for human Antigen receptor, TNF-10, FTZ1 and β-actin.

| hAR-F | GGT TAC ACC AAA GGG CTA GAA |
| --- | --- |
| hAR-R | GAC TTG TAG AGA GAC AGG GTA GA |
| hTNFSF10-F | CAG AGA GTA GCA GCT CAC ATA AC |
| hTNFSF10-R | CCT TGA TGA TTC CCA GGA GTT T |
| hFZD5-F | GTT CGC CAC CTT CTG GAT AG |
| hFZD5-R | AAG CGT TCC ATG TCG ATG AG |
| h-b-actin-F | GGA CCT GAC TGA CTA CCT CAT |
| h-b-actin-R | CGT AGC ACA GCT TCT CCT TAA T |
