# Supplementary material for: Systematically understanding the immunity leading to CRPC progression
Source: PLoS Comput Biol. 2019 Sep 10;15(9):e1007344. doi: 10.1371/journal.pcbi.1007344 (PMC6754164; doi:10.1371/journal.pcbi.1007344)
Supplement: S2 Data — (DOCX) [file pcbi.1007344.s027.docx]

**S2 Data.** The experimental observation and *in silico* prediction of HMSM model.

|  | **Time points**  **(or conditions)** | **Experimental**  **Observation** | ***In silico* Prediction** |
| --- | --- | --- | --- |
| Fold TAM Population | Castrated (day 7) | 1.83$\pm$0.6 | 1.8262$\pm$0.2002 |
| Fold TAM Population | Castrated (day 14) | 2.97$\pm$1.7 | 2.8908$\pm$0.3533 |
| Fold CSF1 Expression | Castrated (day 2) | 2.115$\pm$0.046 | 2.4686$\pm$0.0723 |
| Fold CSF1 Expression | Castrated (day 35) | 4.8$\pm$0.2 | 6.863$\pm$0.7256 |
| Fold IL10 Expression | Castrated (day 2) | 3.0$\pm$0.1 | 2.982$\pm$0.1709 |
| Fold VEGF Expression | Castrated (day 2) | 1.989$\pm$0.536 | 1.5358$\pm$0.0778 |
| Fold TAM population  (CX+PLX VS. CX) | Castrated (day 7) | 0.207$\pm$0.0741 | 0.2092$\pm$0.0631 |
| Fold VEGF Expression  (CX+PLX VS. CX) | Castrated (day 14) | 0.305$\pm$0.0056 | 0.2556$\pm$0.0473 |
| Fold Treg Population in Lymph node | Castrated (2.5 WK) | 1.364$\pm$0.125 | 1.4435+0.3224 |
| Fold Treg Population in Lymph node | Castrated (5 WK) | 1.91$\pm$0.35 | 2.3179$\pm$0.7125 |
| Fold Treg Population in Lymph node | CX+Anti-IL2 VS. CX | 0.4409$\pm$0.107 | 0.4511$\pm$0.1867 |
| Fold CD8+ Population in tumor | Castrated (2.5 WK) | 2.05$\pm$0.25 | 2.1071$\pm$0.7748 |
| Fold CD8+ Population in tumor | Castrated (5 WK) | 1.75$\pm$0.125 | 1.7606$\pm$0.8137 |
| Fold Treg Population in tumor | Castrated (2.5 WK) | 1.7$\pm$0.5 | 2.1094$\pm$0.517 |
| Fold Treg Population in tumor | Castrated (5 WK) | 3.2$\pm$0.6 | 3.2698$\pm$1.0158 |
| Fold tumor growth | Castrated (day 14) | 0.6$\pm$0.1 | 0.5649$\pm$0.0677 |
| Fold tumor growth | Castrated (day 35) | 3.2$\pm$0.3 | 3.1778$\pm$0.4769 |
| Fold tumor growth  (CX+PLX VS. Pre-castration) | Castrated (day 16.5) | 0.5$\pm$0.05 | 0.5082$\pm$0.0393 |
| Fold tumor growth  (CX+PLX VS. Pre-castration) | Castrated (day 35) | 1.5$\pm$0.2 | 1.7745$\pm$0.1839 |
| Fold tumor growth  (CX+Anti-EGFR VS. CX) | Castrated (day 21) | 0.649+0.027 | 0.7667+0.128 |
| Fold tumor growth  (CX+Anti-EGFR VS. CX) | Castrated (day 35) | 0.9+0.1333 | 0.859+0.157 |

CX: Castration

PLX: CSF1R inhibitor PLX3997

The top part of the table was described in “Model evaluation”

The middle part of the table was described in “Model validation”

The bottom part of the table was described in “Model application”
